# In-Depth Proteome Analysis Highlights HepaRG Cells as a Versatile Cell System Surrogate for Primary Human Hepatocytes

**DOI:** 10.3390/cells8020192

**Published:** 2019-02-21

**Authors:** Georg Tascher, Audrey Burban, Sandrine Camus, Marine Plumel, Stéphanie Chanon, Remy Le Guevel, Valery Shevchenko, Alain Van Dorsselaer, Etienne Lefai, Christiane Guguen-Guillouzo, Fabrice Bertile

**Affiliations:** 1 Laboratoire de Spectrométrie de Masse BioOrganique, CNRS, IPHC UMR 7178, Université de Strasbourg, F-67087 Strasbourg, France; marine.plumel@gmail.com (M.P.); vandors@unistra.fr (A.V.D.); 2 Institute of Biochemistry II, Goethe University Hospital, D-60590 Frankfurt am Main, Germany; tascher@med.uni-frankfurt.de; 3 INSERM U1241 NuMeCan, Université de Rennes 1, F-35033 Rennes, France; burban.au@gmail.com (A.B.); christiane.guillouzo@univ-rennes1.fr (C.G.-G.); 4 Biopredic International, Parc d’Affaires de la Bretêche, F-35760 St Grégoire, France; sandrine.camus@biopredic.com (S.C.); valery.shevchenko@biopredic.com (V.S.); 5 CarMeN Laboratory, INSERM, INRA, University of Lyon, F-69310 Pierre-Bénite, France; stephanie.chanon@univ-lyon1.fr (S.C.); etienne.lefai@inra.fr (E.L.); 6 ImPACcell platform, Biosit, Université de Rennes 1, F-35043 Rennes, France; remy.leguevel@univ-rennes1.fr

**Keywords:** Hepatocytes, liver cell lines, HepaRG cells, proteome, secretome, hepatic phenotype, detoxification, liver metabolism, liver diseases, transdifferentiation

## Abstract

Of the hepatic cell lines developed for in vitro studies of hepatic functions as alternatives to primary human hepatocytes, many have lost major liver-like functions, but not HepaRG cells. The increasing use of the latter worldwide raises the need for establishing the reference functional status of early biobanked HepaRG cells. Using deep proteome and secretome analyses, the levels of master regulators of the hepatic phenotype and of the structural elements ensuring biliary polarity were found to be close to those in primary hepatocytes. HepaRG cells proved to be highly differentiated, with functional mitochondria, hepatokine secretion abilities, and an adequate response to insulin. Among differences between primary human hepatocytes and HepaRG cells, the factors that possibly support HepaRG transdifferentiation properties are discussed. The HepaRG cell system thus appears as a robust surrogate for primary hepatocytes, which is versatile enough to study not only xenobiotic detoxification, but also the control of hepatic energy metabolism, secretory function and disease-related mechanisms.

## 1. Introduction

The liver is essential for the maintenance of whole body homeostasis. Many of its fundamental functions are controlled by liver-specific transcription factors during differentiation, and multiple signaling pathways act together for making up the peculiar metabolic behaviors that characterize liver cells [1].

Hepatocytes represent the major cell type of the parenchyma in the liver lobule, and freshly isolated cells are regarded as the gold standard model for studying liver-specific functions [2]. Numerous large-scale omics-based studies have characterized the metabolic signatures of primary human hepatocytes (PHH) [3,4,5], highlighting the intercellular variations that are associated with the complex functional zonation within liver lobules [6]. Today, this metabolic signature is an inescapable reference for many biological and biomedical applications. However, PHH show huge variability in cell activity from one donor to another [7], and the intra-donor heterogeneity (between vials from the same donor preparation) is also high in relation to zonation, lipid content, and cell isolation methods. The availability of these cells remains poor and unpredictable, which further challenges their use for research purposes [8].

Development of alternative hepatic cell models became a main concern during the last decades. Today, among several established cell lines, HepaRG cells have emerged as the most appropriate surrogate to PHH [9]. In contrast to the HepG2 line for example, HepaRG cells greatly benefit from the master and working banks that have been developed early while setting the cell line, thus enabling the reduction of the time of use and of the number of population doublings that are known to be associated with genome deregulation [10]. As a result, HepaRG cells maintain expression of the main hepatocyte functions in good accordance with the regulations that are described in normal PHH, even 20 years after establishment of the line [9]. However, at any time, important variations can occur, depending on the possibly unsuitable procedures that are carried out by users, which can hamper the differentiation process from being fully accomplished. Because of these possible biases and the rapid increase in the number of users worldwide, the need for establishing a reference functional status of differentiated HepaRG cells is crucial. Several transcriptomic [7,11], but only few proteomic [12] analyses have characterized differentiated HepaRG cells, and these studies were essentially focused on the detoxification function. Here, our goal was to take advantage of the powerfulness of deep proteome analysis to delineate the hepato-specific functions harbored by cryopreserved differentiated HepaRG cells commercialized as “HPR116”. These cells are still produced from the early biobanked passages established in 2002 [13], which is large enough to ensure availability of similar HPR116 cells for decades. The use of the quantitative “Total Protein Approach” [14] to estimate protein copy numbers enabled us to compare the structural and functional features of HepaRG cells to those of HepG2 and PHH cells reported in an elegant previous study using the same method [5]. The hepatocyte-like phenotype of the differentiated HepaRG cells was further supported by functional tests, defining the structural and functional bases of HepaRG cells prepared from the early biobanked passages. Our data not only highlight that the HepaRG system is a functionally versatile surrogate model to human hepatocytes, but it also provides a reference for their proteome, their secretome, and their main functions. This will enable the verification of their phenotype in the future.

## 2. Materials and Methods

The evaluation of the mitochondrial activity, bile acid transport and trafficking, and response to insulin of HepaRG cells, and the assays of glycogen storage and fetuin A release are detailed in Supplementary Materials and methods.

### 2.1. Reagents

Unless otherwise specified, all chemicals and reagents were purchased from Sigma Aldrich (St. Louis, MO, USA).

### 2.2. Cell Cultures

HepaRG cells derive from hepatocholangiocarcinoma developed in a female patient suffering from hepatitis C infection. The cells (#HPR116-TA08; Biopredic International, St Grégoire, France) used for expansion came from the original bank established by Gripon et al. in 2002 [13]. Although seeding without basement coating remains suitable for HepaRG cell cultures, collagen coating was used here for all biological assays to favor cell attachment, spreading, and viability. Seeding was performed at a density of 2.6 × 10^4^ cells/cm^2^ in Williams’ E medium supplemented with 2 mM Glutamax, 100 U/mL penicillin, 100 µg/mL streptomycin, 10% fetal calf serum (FCS), 5 µg/mL insulin, and 50 µM hydrocortisone hemisuccinate. The medium was renewed three times a week. After one week, progenitor cells were collected, to evaluate the mitochondrial activity, and to perform release of fetuin A assays and immunocytochemistry analyses. After two weeks, confluence was reached, and the HepaRG cells were shifted to the same medium supplemented with 1.7% of dimethyl sulfoxide (DMSO), hereafter referred to as the differentiation medium, for two additional weeks, leading to confluent differentiated cultures containing equal proportions of hepatocyte-like and progenitors/primitive biliary-like cells. These differentiated hepatic cell cultures were used for preparing HPR116. HPR116 cells correspond to highly differentiated HepaRG cells distributed as frozen vials of 8 million cells by Biopredic International (St Grégoire, France). They were re-seeded at high density (480,000 cells/well of 24-well plates) according to the manufacturer’s recommendations, maintained in long-term culture in the differentiation medium, and used as differentiated hepatic cells after 6, 12, or 18 days of culture for phenotype characterization, except from using proteomics. For proteomics analyses, serum concentration was reduced step-wise over two days (from 10% to 2%) after seeding of HPR116 cells. Then, the medium was changed to a defined serum-free medium containing 0.5% DMSO, and complemented with human Hepatocyte Growth Factor (HGF; 10 ng/mL) and mouse Epidermal Growth Factor (EGF; 2 ng/mL) according to Klein et al. [15], who showed that growth factor supplementation is crucial to maintain the differentiated state and the functional stability of the cells. The cells were maintained for two days in this defined serum-free medium before starting the experiment. Cells were cultivated for 2, 8, and 14 days before the collection of culture supernatants and cell lysis, which corresponded to 6, 12, and 18 days after seeding, i.e., periods where the HepaRG (HPR116) cells were well-differentiated.

Human hepatocytes, distributed as frozen vials of 8 million cells, were obtained from Biopredic International (#HPR116-TA08, St Grégoire, France) in compliance with French bioethics legislation. They were isolated by collagenase perfusion of histologically normal liver fragments from donors undergoing resection for primary and secondary tumors. Two runs of centrifugation and the use of a Percoll gradient allowed for the enrichment of hepatocytes to reach up to 80–90%. The cells were suspended in Williams’ E medium supplemented as above. To ensure low contamination level of these hepatocyte-rich cell populations by non-parenchymal cells, they were seeded at high density, a condition that strongly limits any cell growth during culture. HepG2 cells, distributed as frozen vials of 2 million cells, were purchased from ATCC (#HB-8065, LGC Standards, Molsheim, France). They were cultured in Eagle’s Minimum Essential Medium (EMEM, #30-2003, LGC Standards, Molsheim, France) supplemented with 10% fetal bovine FCS.

### 2.3. Protein Content and Cell Size of HepaRG, PHH and HepG2

After thawing or detachment and cell counting, cell suspensions were centrifuged, and the pellets were lysed with 0.05 N NaOH at 37 °C for 1 h. Protein concentration was determined with a BCA protein assay (Pierce Biotechnology, Rockford, IL, USA) kit according to the manufacturer’s instructions.

Cell sizes of living cells in suspension in corresponding media, were determined for the three cell systems from two different batches each (two replicates for each batch) by phase contrast microscopy, using an inverted microscope (Olympus, Waltham, MA, USA). Cell volume was calculated, assuming a spherical shape based on the cells’ average radii.

### 2.4. Karyotyping of HepaRG and HepG2 Cells

Subconfluent living cells of HepG2 and HepaRG were used. The analysis was performed at the CERBA laboratory (Medical Genetics Oncohematology, Department of Human Genetics, Paris, France).

### 2.5. Immunocytochemistry Performed on HepaRG Cells

Cells were washed with warm phosphate buffered saline (PBS), fixed with either methanol for 15 min at −20 °C, or with 4% paraformaldehyde for 20 min. They were then washed three times with cold PBS. After paraformaldehyde fixation, cells were permeabilized for 20 min with 0.3% Triton (Sigma Aldrich, St. Louis, MO, USA) in PBS, followed by 1 h of incubation in PBS containing 1% bovine serum albumin and 5% normal donkey serum. Cells were then incubated overnight with primary antibodies (see Table S1) targeting the zona occludens protein-1 (ZO-1), HNF4, and β-catenin diluted in PBS containing 1% bovine serum albumin and 5% normal donkey serum. After washing with cold PBS, the cells were incubated for 2 h with mouse- or rabbit Alexa fluor-labeled secondary antibodies (Invitrogen, ThermoFisher Scientific, Rockford, IL, USA, Table S1). Finally, cells were again washed with cold PBS and incubated for 20 min. with Hoechst dye (#H6024; diluted 1/1000; Sigma Aldrich, St. Louis, MO, USA) and rhodamine–phalloidin fluoroprobe SR101 (#R415; 200 U/mL; diluted 1/100; ThermoFisher Scientific/Life technology; Carlsbad, CA, USA) in PBS for 20 min for nuclei and F-actin labeling, respectively. Immunofluorescence images were detected by using a Cellomics ArrayScan VTI HCS Reader (ThermoFisher Scientific, Rockford, IL, USA).

### 2.6. Mass Spectrometry Analyses Performed on the HepaRG Cell System

#### 2.6.1. Protein Extraction

HP116 cells were cultivated in 6-well plates under serum-free conditions, with medium renewal every two days, as previously described [15]. After 6, 12, and 18 days, culture supernatants containing secreted proteins from one well per time-point were collected and cells were washed twice with warm (37 °C) and twice with cold (4 °C) PBS before lysis in the culture plates using a buffer containing 50 mM Tris pH 7.5, 150 mM NaCl, 1 mM EDTA, 0.1% (*w*/*v*) *N*-lauryl sarcosine and a protease inhibitor cocktail. After 30 min at 4 °C, cells were scraped off the plates using a rubber-policeman (Sarstedt, Nümbrecht, Germany), and the cell debris was pelleted by centrifugation at 5000× *g* for 10 min at 4 °C before storage of the supernatant at −80 °C. For secretome analysis, cell culture supernatants were cleared by centrifugation (1000× *g* for 5 min at 4 °C), filtered using 0.45 µm syringe filters (Minisart, Sartorius, Dourdan, France) and, following the addition of a protease inhibitor cocktail, they were snap-frozen in liquid nitrogen and stored at −80 °C before further processing.

#### 2.6.2. Sample Preparation for Intracellular Proteome Analysis

Intracellular protein extracts from three different days were pooled and protein concentration was estimated by a Bradford assay using BSA as standard (Bio-Rad, Hercules, CA, USA). Five aliquots of a volume corresponding to 50 µg of proteins was precipitated with 10% trichloroacetic acid (TCA) overnight at 4 °C. After centrifugation (10 min at 13,000× *g*), protein pellets were washed with tetrahydrofuran (3 × 400 µL) in order to remove the remaining salts and the co-precipitated sarkosyl. Three of the aliquots were digested in-solution and the remaining two aliquots were in-gel digested after prefractionation, using sodium dodecyl sulfate polyacrylamide gel electrophoresis (SDS-PAGE) as described below. For liquid digestion, proteins were solubilized in 200 µL of denaturation buffer (8 M urea, 100 mM ammonium bicarbonate), disulfide bonds were reduced with 12 mM dithiotreithol (30 min at 37 °C) and free sulfhydryl groups were alkylated using 40 mM iodoacetamide over one hour at room temperature in the dark. Afterwards, samples were diluted with digestion buffer (100 mM ammonium bicarbonate) to reduce the concentration of urea to below 1 M, and proteins were digested with trypsin (sequencing-grade; Promega, Madison, WI, USA) overnight at 37 °C, applying a trypsin-to-protein ratio of 1:100. Trypsin activity was quenched by the addition of formic acid to a final concentration of about 0.5% (pH < 2), and peptides were cleaned-up using solid phase extraction (SPE) cartridges (SEP-PAK Vac tC18, 1 cc, 50 mg; Waters, Milford, MA, USA). Sample volume and organic solvent concentration were reduced using a vacuum centrifuge (Speed vac Savant; Thermo Scientific, San Jose, CA, USA) and samples were adjusted to 100 µL with 0.1% formic acid in water before MS (mass spectrometry)-analysis. For prefractionation on the protein level, precipitated proteins were solubilized in SDS-PAGE sample buffer (2.5% SDS, 10% glycerol, 5% β-mercaptoethanol, and 0.1% bromophenol blue in 50 mM Tris) and separated on a 12% polyacrylamide gel until they had migrated 12 cm into the separation gel. Lanes were cut into six bands of equal size following Colloidal Coomassie Blue (#G250, Sigma Aldrich, St. Louis, MO, USA) staining and subsequently de-stained. Proteins were reduced and alkylated using a robotic pipetting device (MassPrep Station; Waters, Milford, MA, USA) as previously described [16]. For in-gel digestion, 75 ng of sequencing grade trypsin (Promega, Madiso, WI, USA) was added to each band and incubated for 16 h at 37 °C. Peptides were extracted in 40% acetonitrile (ACN), 0.1% formic acid, followed by another extraction step with 60% ACN, 0.1% formic acid for 1 h at 450 rpm on an orbital shaker, and samples were stored at −80 °C until LC-MS analysis. Prior to injection, peptide extracts were evaporated in a vacuum centrifuge (ThermoFisher Scientific, Rockford, IL, USA), and resuspended in 2% ACN with 0.1% formic acid.

#### 2.6.3. Sample Preparation for Secretome Analysis

Culture supernatants of day 6, 12, and 18 were pooled and three aliquots of 2 mL each were concentrated using ultrafiltration (MWCO 5 kDa, Vivaspin4; Sartorius, Dourdan, France) over 1 hr at 4000× *g* at 4 °C. Buffer was exchanged twice by adding 3 mL of PBS, and centrifugation, as described above. For specific depletion of albumin, 180 µg of proteins were incubated with antibody-coated sepharose beads (Proteome purify 2; R&D systems, Lille, France) for one hour at 4 °C, with subsequent filtration using the spin-filter devices contained in the kit to obtain the Albumin-depleted fraction. Bound albumin was eluted from the resin with 500 µL of 200 mM Glycine, pH 2.8 and protein concentrations were estimated before and after depletion by using a Bradford assay (Bio-Rad). A total of 45 µg of depleted sample was digested in-solution, as described for the intracellular proteome, using a trypsin-to-protein ratio of 1:90.

#### 2.6.4. Mass Spectrometry Analysis of the Intracellular Proteome

Peptide mixtures were analyzed on a nanoACQUITY UPLC system (Waters, Milford, MA, USA) coupled to an Impact-HD quadrupole-time-of-flight (Q-TOF) mass spectrometer equipped with a Captive Spray ion-source and a nanoBooster (Bruker Daltonics, Bremen, Germany). The platform was controlled via Hystar (v 3.2; Bruker Daltonics, Bremen, Germany) and OtofControl (Rev 3.4; Bruker Daltonics, Bremen, Germany). The solvent system consisted of 0.1% formic acid in water (solvent A) and 0.1% formic acid in acetonitrile (solvent B). Peptides (1 µg) were first desalted on a precolumn (Acclaim PepMap 100, 100 µm × 2 cm, 5 µm; ThermoFisher Scientific, Rockford, IL, USA) at 1% B, at a flow rate of 10 µL/min for 10 min, and then separated using a 180 min gradient from 1 to 43% B at 450 nL/min, using a 50 cm column (Acclaim PepMap C18, 50 cm × 75 µm, 2 µm; ThermoFisher Scientific, Rockford, IL, USA) held at 60 °C in a column oven (Applied Biosystems, ThermoFisher Scientific, Rockford, IL, USA). The mass spectrometer was operated in positive mode using the following settings: source temperature set to 150 °C, dry gas flow set to 3 L/min, and spray voltage optimized to 1300 V. Acetonitrile was used as a dopant in the nanoBooster (Bruker Daltonics, Bremen, Germany), and the nebulizer pressure was set to 0.2 bar. Spectra were acquired by automatic switching between MS and MS/MS modes in a mass range of 100–2200 *m*/*z*, with a fixed cycle time of 3.5 s. The MS acquisition rate was set to 2.5 Hz, and the MS/MS acquisition rate ranged from 3 to 30 Hz, depending on the precursor intensity. Preferably, ions with a charge of 2 to 5 were selected for *c*ollision-induced dissociation (CID) fragmentation, using nitrogen as the collision gas. Ions were excluded from fragmentation after the acquisition of one MS/MS spectrum, and exclusion was released after 0.5 min, but only if the precursor intensity did not exceed the value during the first selection by a factor of three. If this was the case, the precursor was fragmented again to increase the MS/MS spectra quality. Online correction of TOF calibration was performed using hexakis(2,2,3,3,-tetrafluoropropoxy)phosphazine ([M + H]+ 922.0098 *m*/*z*) as the lock-mass, which was spiked into the dopant solvent and thus continuously delivered together with the nebulizer gas. Each of the three liquid digest and the two in-gel digest replicates were analyzed twice (injection duplicates).

#### 2.6.5. Mass Spectrometry Analysis of the Secretome

LC-MS/MS analysis of the tryptic peptides derived from the depleted secretome samples was performed essentially as described above for the intracellular proteome, except different types of columns were used for the analyses. Peptides (500 ng) were loaded at 1% B at a flow rate of 5 µL/min for 3 min onto the precolumn (BEH C18, 180 μm × 20 mm, 5 µm; Waters, Milford, MA, USA), and then separated using a 60 min gradient from 5–35% B on a 25 cm column (BEH C18, 25 cm × 75 µm, 1.7 µm; Waters, Milford, MA, USA). Each of the three depletion replicates were analyzed three times (injection triplicates).

#### 2.6.6. Mass Spectrometry Data Processing

MS raw data processing was performed in MaxQuant (v. 1.5.3.30; Max Plank Institute of Biochemistry, Martinsried, Germany) with default parameters if not specified otherwise. Using the Andromeda search engine [17], peak lists were searched against human protein sequences (Taxonomy ID 9606; 20194 sequences) downloaded from SwissProt [18] using the MSDA software suite [19], to which sequences of common contaminants (247 entries) were added automatically by MaxQuant (v. 1.5.3.30; Max Plank Institute of Biochemistry, Martinsried, Germany). Identifications were filtered to obtain false discovery rates (FDR) of below 1% for both peptide spectrum matches (PSM; minimum length of seven amino acids) and proteins, using a target-decoy strategy. The “match between runs” option was enabled, in order to transfer identifications from one run to another, using a match time window of 0.5 min after retention time alignment, based on an alignment time window of 10 min, keeping unmodified counterpart peptides for quantification. For the extracellular proteome (“Secretome”), only proteins that had been identified in at least one of the three injection replicates in all three depletion replicates were kept for further analyses.

The mass spectrometry proteomics data from HepaRG cells were deposited to the ProteomeXchange Consortium via the PRIDE [20] partner repository, with the dataset identifier PXD010304.

#### 2.6.7. Mass Spectrometry Data Analysis

Protein copy numbers and secretion rates were calculated using the “Total Protein Approach” [14], based on raw spectral intensities, and by scaling to the total protein content per cell of the three different cell types, as determined in the course of this study. All other data analyses and visualizations was performed in Perseus and R [21], using the Normalyzer package [22], including a global LOESS normalization of calculated protein copy numbers, to account for the increased proteome depth of the data set of Wisniewski et al. [5]. We intentionally did not use the recently described “histone ruler” [23], because of “atypical” DNA contents in these cell types, since PHH are partially multinuclear [24] and the other two cell types showed abnormal karyotypes (see the main text), potentially leading to biases in copy number estimations.

Proteins of given pathways were categorized on the basis of their presence in canonical pathways or other networks contained in the Ingenuity® Pathway Analysis database [25]. For protein categorization, pathway maps from the Kyoto Encyclopedia of Genes and Genomes database [26] were also used, as well as the automatic extraction of Gene Ontology annotations using the MSDA software [19] and literature examination.

Regarding the proteins identified in the HepaRG secretome, bona-fide secreted proteins were recognized using the SignalP 4.1 [27] and SecretomeP 2.0 [28] algorithms. Hence, secreted proteins are those containing a signal peptide for entering the classical secretory pathway, or those predicted to be secreted via alternative secretion routes. The remaining proteins were still considered to be secreted, if they were annotated to be localized to the extracellular space or to exosomes. Protein secretion rates were calculated by using the “total protein approach” based on total protein in the depleted sample normalized to the number of seeded cells and days of secretion into the medium before medium collection (i.e., 2). The secretion rate of albumin was calculated by using protein loss during depletion, and by normalizing this value to the purity of albumin in the bound fraction assessed by SDS-PAGE, and by subsequent analysis of gel scans after Coomassie Blue staining using Image J v1.51j8 [29].

#### 2.6.8. Comparison of the Intracellular Proteome of the Three Cell Systems

PHH and HepG2 data were generated as described in Wisniewski et al. [5]. In brief, PHH from seven donors were prepared in duplicate, except for two donors, which were analyzed once, while cell lysates of HepG2 cells were prepared in triplicate. As reported above for HepaRG cells, a mixture of cell lysates from one six-well plate on days 6, 12, and 18 was analyzed in five replicates, two of them digested in-solution, and three digested in-gel after prefractionation by SDS-PAGE. All samples were injected twice as technical replicates, from which the mean was calculated for further data analysis. The quantitative comparison of protein abundances (copy numbers) in the three cell systems was assessed statistically in the R software environment (v. 3.4.0, The R Foundation for Statistical Computing, Vienna, Austria) [21], using 1-way analysis of variance (ANOVA) with Tukey as post hoc tests, including the adjustment of p-values according to Bonferroni–Holm. For selected pathways or signaling cascades, heatmaps showing the relative differences of protein abundances (copy numbers) in HepaRG and HepG2 cells versus PHH were built using R (v3.0.2, The R Foundation for Statistical Computing, Vienna, Austria). In addition to the consideration of statistical significance, we decided to specifically discuss the obvious differences, i.e., the cases where proteins are absent–present, depending on the cell system or when they exhibited fold-changes that exceeded a value of 20. In the 2D annotation enrichment, scores were calculated by using a MANOVA test as described earlier [30].

#### 2.6.9. Batch-to-Batch Stability of the Proteome of Differentiated HepaRG Cells (HPR116)

Three different batches of cryopreserved differentiated HepaRG cells were cultivated in quadruplicates (n = 4), according to manufacturer’s instructions in 6-well plates, and proteins were extracted as described for the analysis of the intracellular proteome used for copy number estimations. Protein concentrations in the cell extracts were estimated by a Bradford assay, using BSA as a standard (both Bio-Rad, Hercules, CA, USA), and 20 µg of proteins were precipitated by adding nine volumes of chilled acetone, with incubation for 3 h at −20 °C. Precipitated proteins were pelleted by centrifugation (15 min at 16,000× *g*), resuspended in SDS-PAGE sample buffer (2.5% SDS, 10% glycerol, 5% β-mercaptoethanol, and 0.1% bromophenol blue in 50 mM Tris), and separated on a 12% polyacrylamide gel. Lanes were cut into six bands following colloidal Coomassie blue staining, and subsequently de-stained as well as reduced and alkylated, using a robotic pipetting device (MassPrep Station; Waters, Milford, MA, USA). For in-gel digestion, 75 ng of sequencing-grade trypsin (Promega, ThermoFisher Scientific, Rockford, IL, USA) were added to each band and incubated for 16 h at 37 °C. Peptides were extracted as described above, and the samples were stored at −80 °C until LC-MS analysis. Prior to injection, peptide extracts were evaporated in a vacuum centrifuge (ThermoFisher Scientific, Rockford, IL, USA) and resuspended in 12 µL 2% ACN with 0.1% formic acid.

LC-MS analyses were performed using an Agilent 1100 series nanoLC-Chip/MS system (Agilent Technologies, Palo Alto, CA, USA) coupled to an amaZon ion trap (BrukerDaltonics, Bremen, Germany). The system was fully controlled by HyStar 3.2 (BrukerDaltonics, Bremen, Germany). The chip contained a Zorbax 300SB-C18 column (43 mm × 75 µm, 5 µm particle size; Agilent Technologies, Palo Alto, CA, USA) and a Zorbax 300SB-C18 enrichment column (40 nL, 5 µm particle size; Agilent Technologies, Palo Alto, CA, USA). The solvent system consisted of 2% acetonitrile, 0.1% formic acid in water (solvent A), and 2% water, 0.1% formic acid in acetonitrile (solvent B).

A total of 5 µL of each sample were loaded onto the enrichment column at a flow rate set to 3.75 µL/min, with solvent A. Elution was performed at a flow rate of 300 nL/min, with a 8–40% linear gradient (solvent B) in 60 min, followed by a 4 min stage at 70% of solvent B, before reconditioning the column at 8% of solvent B. MS spectra were acquired with the following settings: source temperature was set to 135 °C while cone gas flow was at 3 L/min. The nanoelectrospray voltage was optimized to −1850 V. MS spectra were acquired in the positive ion mode in a mass range of 250 to 1500 *m*/*z*, using standard enhanced resolution at a scan rate of 8100 *m*/*z*/s. Ion charge control was fixed at 200,000 with a maximum injection time of 200 ms. For tandem MS experiments, the system was operated with automatic switching between MS and MS/MS modes. Fragmentation was performed by using argon as the collision gas. The eight most abundant precursors were selected on each MS spectrum for further isolation and fragmentation, with a preference for doubly-charged ions (absolute threshold of 5000, relative threshold 5%). Ions were excluded after the acquisition of one MS/MS spectra, and the exclusion was released after 0.3 min. The Smart Parameters Setting option was used for the selected precursor ions. The MS/MS spectra were acquired using a mass range from 100 to 2000 *m*/*z*. The Ion Charge Control was fixed at 400,000 and two scans were averaged to obtain a MS/MS spectrum.

Peak lists in Mascot generic format (.mgf) were generated using Data Analysis (version 4.0; Bruker Daltonics, Bremen, Germany), and searched against a SwissProt-derived [18] combined target-decoy database (created on 31 July 2012, containing 20,250 target sequences, plus the same number of reversed decoy sequences) using Mascot (version 2.4.1; Matrix science, London, United Kingdom). The database contained sequences of human proteins including common contaminants (human keratins and porcine trypsin) and was created using an in-house database generation toolbox [19]. During the database search, up to one missed cleavage by trypsin and two variable modifications (oxidation of methionine (+16 Da) and carbamidomethylation of cysteine (+57 Da) were considered. The search window was set to 0.25 Da for both the precursor and fragment ions. Mascot result files (.dat) were imported into Scaffold 3 software (v3.6.5; Proteome Software Inc., Portland, OR, USA) and probability-based scoring of the identified peptides were taken into account to avoid the consideration of poor quality data. Hence, only proteins with an ion score of above 25 and an ion minus identity score of at least 2 were accepted resulting in a protein false discovery rate (FDR) of below 1%, based on the number of decoy hits. For the determination of significantly changing proteins between groups, only proteins with at least four spectra (taking into account only spectra specific for a given protein group) in at least one of the samples were retained. Multiple and pairwise comparisons of spectral counts, i.e., of relative protein abundances between groups were performed by using the beta binomial and inverted beta binomial tests, respectively [31]. To increase the power of the statistical tests while keeping under control the family-wise Type I error, p-values from pairwise comparisons were corrected using the Holm–Bonferroni method. Proteins with a corrected *p*-value of below 0.05 were considered to be significant. All statistics were performed by using the R package [21]. As an additional criterion, only proteins with a fold-change of more than 2-fold between the compared groups were considered to be differentially abundant.

## 3. Results and Discussion

### 3.1. Main Characteristics of Highly Differentiated HepaRG Cells (HPR116)

**Figure 1:**
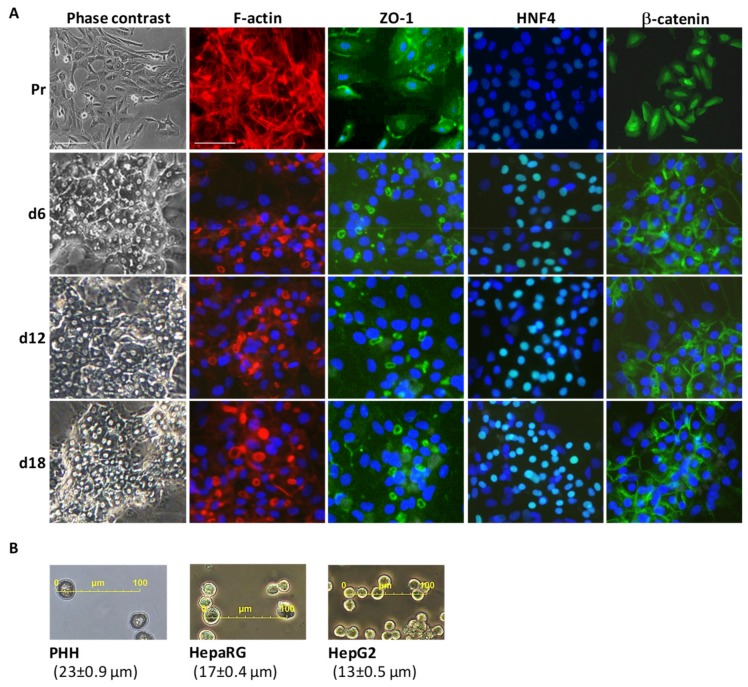
The main features of highly differentiated HepaRG cells cultivated in the differentiation medium (1.7% dimethyl sulfoxide (DMSO), 10% fetal calf serum (FCS)). (**A**) Phase contrast microscopy, staining of cytoskeletal F-actin, and the immunostaining of ZO-1 junctional protein, HNF4 transcription factor, and β-catenin in HepaRG progenitors (Pr) and differentiated HepaRG (HPR116) cells at days 6, 12, and 18 (d6 to d18). (**B**) Mean cell size (± SD) of primary human hepatocytes (PHH) and HepaRG and HepG2 cells (see also Table S2).

HPR116 is a source of highly differentiated HepaRG cells, and large batches can be produced very conveniently. In the culture conditions described in the Methods section, frozen differentiated cells are seeded at confluence. They reorganize in 3–4 days in typical colonies of hepatocytes, surrounded by primitive biliary cells [13]. After day 6, their differentiated state remains stable for at least two weeks (Figure 1A). In comparison with the undifferentiated features of the progenitors from the proliferative stage, the distribution of cytoskeletal filaments and the junctional ZO-1 protein is restored in HPR116 at day 6, hence providing support for cell polarity and the appearance of bile canaliculi at the biliary pole. At this stage, and as expected in normal mature hepatocytes HNF4 and beta-catenin, two key factors that were known to control the liver differentiation program, are localized in hepatocyte nuclei and translocated to the plasma membrane, respectively. Cells and media from day 6 to 18 thus appear to be representative of the high functional capacity of HepaRG (HPR116) cells. These results are in good accordance with a previous study reporting that HPR116 maturation and differentiation dynamics stabilize ~7 days after cell plating [32].

Using microscopy to measure the sizes of the cells, we first observed that the HepaRG cells were slightly smaller than PHH, and that HepG2 cells are even smaller (Figure 1B and Table S2). As a result, despite the lower absolute amounts of total protein per cell, the intracellular protein concentration is much higher in the HepG2 line. Since the HepG2 cell line was established in 1976 [33], the cells have drastically evolved and changed (Table S3), passage after passage; e.g., regarding the stability of their chromosomal profiling and ploidy [34], and, unlike HepaRG, no early biobanked HepG2 cell passages are available today. Accordingly, we observed trisomy 2 and 10, tetrasomy 16 and 20, and additional aberrations that were markedly variable, even within the same culture dish (Figure S1). In contrast, only trisomy 7 and a translocation between chromosomes 12 and 22 were consistently observed in HPR116 cells (Figure S1).

Reproducibility across different batches is an indispensable prerequisite for the use of a given cell model. We confirmed the great reproducibility (low batch-to-batch variation) of HepaRG (HPR116) cells at the proteomic level, with the abundance of only 24 of 1155 analyzed proteins being significantly different when comparing the three different batches (N = 4 per batch, Table S4).

HPR116 produces mature human hepatocyte-like cells, are technically easy to cultivate, and can be used with high reproducibility. Using several markers, including CYPs expression, Klein et al. observed that HepaRG cells are highly differentiated and stable at days 6, 12, and 18, with the maximum of differentiation being obtained as early as day 6 [15]. This is in perfect line with the microscopy and immunostaining data described above. Klein et al. also showed that the differentiation patterns of HepaRG cells are very similar when they are cultivated in a defined serum-free medium (0.5% DMSO) supplemented with growth factors that are essential for maintenance of the differentiation state and the functional stability of the cells (see the Methods section) [15]. With such defined serum-free media opening the way to proteomics analyses, we decided to analyze the intracellular and secreted proteomes of a mix of HepaRG cells from days 6, 12, and 18.

### 3.2. Large-Scale Proteomics of Highly Differentiated HepaRG

**Figure 2:**
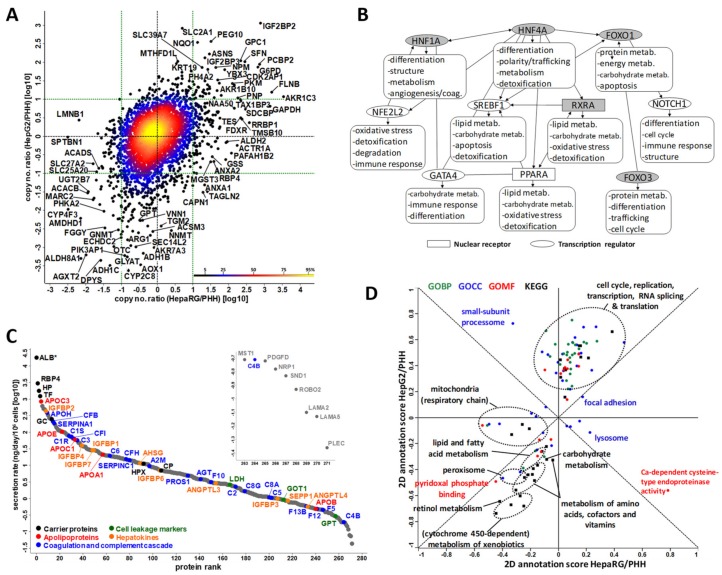
Characterization of the proteome and secretome of highly differentiated HepaRG cells cultivated in defined serum-free medium (0.5% DMSO) supplemented with growth factors. (**A**) Protein copy numbers per cell in HepaRG and HepG2 cells versus PHH (see also Tables S4–5 and Figure S4). (**B**) Master regulators (in grey when not detected) of the hepatic phenotype and the main functions associated with their main (downstream) related factors (see details in Figure S3). (**C**) Secretion rates of the 272 proteins secreted by HepaRG cells. * Extrapolated values for albumin (ALB) after immunodepletion (see the Methods section and Table S5). (**D**) 2D annotation enrichment from 3995 proteins identified in at least two of the three cell types (see also Figure S2 and Table S6).

Overall, 4394 proteins were identified from whole-cell lysates, providing a valuable proteomic resource for the in-depth characterization of differentiated HepaRG cells (Figure 2A and Table S5A). The identified proteins were derived from all cellular compartments, and they covered many biological processes that were related to liver-specific functions (Figure S2A,B). The majority of proteins is associated with metabolism, with a broad representation of carbohydrate and lipid metabolisms, and, as expected, of the detoxification function, including xenobiotic biodegradation, urea, and bile acid metabolism. HepaRG cells are thus well-equipped to express high levels of metabolic activity that are required for functional hepatocytes. Importantly, almost 20% of the identified intracellular proteins were annotated within the ‘protein secretion’ biological process. This is in perfect line with the expected secretory activity of liver-like cells. The secretome profile of HepaRG cells that we established in parallel with their intra-cellular proteome, comprised 313 proteins (Table S5C), of which 272 bona-fide secreted proteins were retained (Figure S2C). These secreted proteins mainly covered the characteristic panel of transport proteins (31%), the proteolytic machinery (10%), the coagulation and complement cascade (17%), and extracellular matrix components (8%) (Figure S2D). Of note, the majority (56%) of the proteins that were not predicted as being secreted were annotated to be located in exosomes, which are important extracellular vesicles that are gaining increasing attention in studies dealing with disease mechanisms (Figure S2C).

Master regulators of the hepatic phenotype encompass major transcription factors (HNF4A, HNF1A, NFE2L2, SREBF1, FOXO1 and FOXO3, GATA4, and NOTCH1) and nuclear receptors (RXRA and PPARA). Using the IPA Network Generation Algorithm [25], we selected the top 50 or top 100 downstream proteins connected (i.e., exhibiting the strongest biological relationships) to these master regulators, and which were well-expressed in HepaRG cells (Figure 2B). Functional categorization showed that the HepaRG data was highly consistent with the main functions driven by hepatic master regulators (see Table S5A and Figure S3). For example, most of downstream SREBF1 and PPARA targets are related to lipid metabolism (43–45%), and those of HNF4A and HNF1A are involved in most aspects of mature hepatocyte functions. In addition, the proteins that are key to hepatocyte-like cells, including for instance, those that ensure cell polarization, trafficking and degradation processes, were also present in the proteome of the HepaRG cells.

Using the “Total protein approach” based on the total protein amount in the cell culture supernatants, we ranked the secreted proteins according to their secretion rates (Figure 2C). Many of the proteins secreted by the HepaRG cells are known to be secreted by hepatocytes, including carrier proteins, apolipoproteins, haptoglobin, coagulation factors, and hepatokines. The calculated secretion rates ranged from subnanograms (for known low-abundance circulating proteins, e.g., PDGFD) to micrograms (for known major blood proteins, like albumin or serotransferrin) per day per million cells, spanning six orders of magnitude in total. Notably, cell leakage markers like LDH, GOT1, and GPT were detected at very low concentrations (<3 ng/day/10^6^ cells) indicating minor contamination of the secretome by intracellular proteins due to cell death. The secretome profile of HepaRG cells established here thus appears to be highly representative of what would be expected from a good cell system surrogate to human hepatocytes, provided that the cells cultivated in the defined serum-free but growth factor-supplemented medium are indeed fully differentiated, as shown by Klein et al. [15].

To better characterize the proteome of the HepaRG cells, we next compared our dataset to those produced previously for HepG2 and PHH cells [5]. The use of similar proteomics strategies and computational methods for establishing protein copy numbers (see the Methods section), and overall quality of the proteomics data (Figure S4A,B) suggested that the comparison of the three cell systems could be done with minimal biases. In addition, the total calculated numbers of protein molecules per cell (Table S2) were in good concordance with theoretical calculations, based on well-established cellular parameters [35]. Hierarchical clustering and 2D annotation enrichment analysis highlighted the HepaRG proteome as being closer to PHH than to HepG2 (Figure S4C,D). When considering the 3995 proteins identified in at least two of the three cell systems, 484 appeared to be absent from HepG2, and 31 from PHH (Table S5B), while the abundance of a comparable number of proteins was significantly different when comparing HepaRG and HepG2 cells to PHH (1790 and 1646, respectively) and HepaRG to HepG2 cells (1598). Based on the ratios of the proteomics-derived copy numbers, we observed that HepaRG and HepG2 cells are consistently more or less equally distant from PHH. It can be seen in Figure 2A that 75% of the proteins exhibited a less than 10-fold difference in copy numbers for the two cell lines versus PHH, with HepaRG being closer to PHH, as illustrated by the red ellipse comprising 50% of the proteins, which appears compressed along the x-axis. Remarkably, the overexpression of IGF2BP2 (~1000-fold), and more generally, of proteins related to the cell cycle and DNA and RNA metabolism, in both HepaRG and HepG2 cells (Figure 2D; see also Table S5) reflected their liver cancer origin. An alternative explanation may be due to the techniques of human hepatocyte isolation/selection, which generally limits the numbers of non-parenchymal cells such as progenitors in PHH cultures, whereas HepaRG cell monolayers contain progenitor-like cells. Finally, several proteins that are involved in lipid and /or carbohydrate metabolism (e.g., ACSM3, ADH1B, ADH1C, ECHDC2, SEC14L2), the urea cycle (OTC, ARG1), the detoxification function (AKR7A3, CYP2C8, GLYAT), and the response to oxidative stress (AOX1) were downregulated in both cell lines relative to PHH, albeit in a less marked manner in HepaRG than in HepG2 (Figure 2A). The reverse was true for very few proteins involved in lipid and/or carbohydrate metabolism (e.g., ACACB, ACADS, SLC27A2, SLC25A20), and in the detoxification function (UGT2B7), which were downregulated in both cell lines relative to PHH, albeit in a less marked manner in HepaRG than in HepG2 cells (Figure 2A).

### 3.3. Structural Hotspots of Highly Differentiated HepaRG

**Figure 3:**
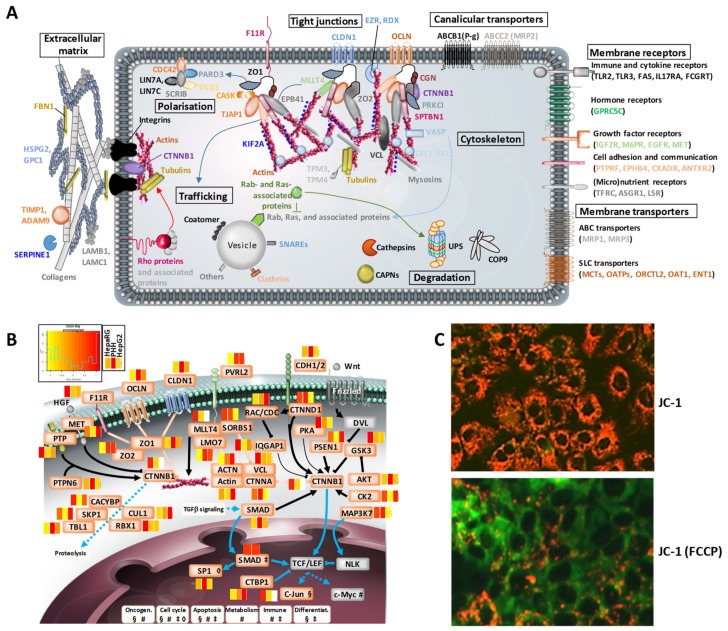
Structural hotspots and signaling pathways that are essential to the hepatic phenotype of highly differentiated HepaRG cells. (**A**) Main structural proteins identified in HepaRG cells cultivated in the defined serum-free medium (0.5% DMSO and growth factor supplementation). See also Tables S5 and S7 for a comparison with HepG2 cells and PHH, and Figure S5 for a zoom on the zonation factors. (**B**) Relative differences of protein abundances in HepaRG (defined serum-free medium) and HepG2 cells versus PHH, shown as heatmaps for the β-catenin signaling pathway (white boxes: proteins not seen). Non-detected proteins are colored in grey. (**C**) Mitochondrial membrane potential (MPT) assessed by JC-1 staining in HepaRG cells cultivated in the differentiation medium (1.7% DMSO, 10% FCS), and treated or not, with or without treatment with the FCCP uncoupler agent; red and green fluorescence indicate hyperpolarization and depolarization, respectively.

The polarized architecture [36] and structural dynamics that characterize a typical mature hepatocyte involve the extracellular matrix, cytoskeleton, tight junctions, and the active dynamic machinery. Based on a literature examination, 355 proteins that are representative of these key elements were selected from our HepaRG proteomics dataset in a non-exhaustive manner, and they were distributed among 10 functional groups which were further divided into protein families (see Table S5A). The key structural elements that characterize a hepatocyte are thus well-expressed in HepaRG cells (Figure 3A).

We found that the expression levels (median copy numbers) of most of the structural proteins were globally similar in the three cell systems (see Tables S5B and S7). Exceptions included several important protein families, which gives clues about the following hepatic-specific structural dynamics below.

i) Contractility and trafficking. Calpains (CAPN1, CAPN2 and CAPNS1) participate in cytoskeleton remodeling, and they mediate signal transduction; their levels are, on average, ~9 times higher in HepaRG than PHH, while being lower in HepG2. Ras-related proteins RAC2, RAB-5C, -6A, -6B, -6C, -27B, and Rab-related GTPase-regulating proteins (RAB Gap1L, RAB3GAP1, TBC1D1, TBC1D2B, TBC1D22A, and TBC1D31) are well-detected in HepaRG, but not in HepG2 cells. The key protein RhoA was also detected only in HepaRG cells, while its main effectors ROCK1, MYL1/6/K and proteins of the Eph/ephrin signaling pathways were expressed at higher levels in HepaRG than HepG2 cells. By interacting with the cytoskeleton, all of these proteins are able to regulate cytoskeletal dynamics, including bile canaliculi rhythmic movements and bile acid efflux [37]. They are also main actors in controlling cell shape and polarity, and signal transduction during differentiation and proliferation [38]. Importantly, the spectrin beta chain SPTBN1 that links actin to the plasma membrane and which triggers calcium-dependent movement of the cytoskeleton, is on average, ~320 times less abundant in HepaRG than in PHH and HepG2 cells. This may have been influenced by the defined serum-free medium that was used for the HepaRG cultures (0.5% DMSO and growth factor supplementation). Such regulation is nevertheless in line with the balanced role of SPTBN1 in acting as i) a “tumor suppressor” in the proliferation of mature hepatocytes and ii) an activator of progenitor cells through the control of transforming growth factor beta signaling [39]. Hence, low expression levels of SPTBN1 could contribute to maintaining progenitor cells, a main characteristic of the HepaRG cell line.

ii) Cell–cell communications and polarity. Proteins forming or that are associated with tight junctions involve the receptor CXADR and cytosolic protein cingulin (CGN), which are linked to the tight junction protein ZO-1, as well as Claudins, MAGUK proteins, and receptor-type tyrosine-protein phosphatase F (PTPRF). Among these proteins, CGN is detected in HepaRG, and not in HepG2 cells; it is essential for the formation and regulation of the tight junction paracellular permeability barrier, and for the delineation of the biliary pole [40]. This function is crucial, and as suggested here, it is effective in HepaRG. Meanwhile, a number of proteins involved in cell adhesion, adherens and tight junctions (e.g., desmoplakin, galectin 4, occludin), and in the polarized organization of the actin fiber network (e.g., plastin 2, MYL12A) were seen to be less abundant in HepaRG cells than PHH (see Table S5). By ensuring the maintenance of plasticity and motility in differentiated HepaRG cells, this may favor their ability to transdifferentiate, i.e., to maintain stemness properties and/or a pool of early progenitor cells in parallel to the differentiation process [10].

iii) Morphogenesis. Beta-catenin (CTNNB1) is a key component of the Wnt signaling pathway and it is involved in the E-cadherin–catenin adhesion complex, together with afadin (MLLT4) and Ephrin type-B receptor 4 (EPHB4), which influences developmental processes in the liver bud. The striking observation is the fact that CTNNB1 and MLLT4 were detected only in PHH and HepaRG cells, with expression levels being higher in the latter (77 and six times, respectively; Tables S5 and S7). Such data, together with the regulation of SPTBN1 levels (see above), would support the contribution of these factors towards the unique transdifferentiation capability of HepaRG cells, a unique property that is yet to be elucidated. Moreover, the fact that CTNNB1 was immunolocalized in the nuclei of HepaRG progenitors/hepatoblasts contrasts with its deposition at the plasma membrane of differentiated cells (see Figure 1). This illustrates that the regulation of the transduction signaling process and of the spatiotemporal expression of this protein is normal and comparable to that in PHH [41]. In HepG2 cells, CTNNB1 was not identified by mass spectrometry, which may be due to the fact that its sequence is known to be mutated in these cells, with, for example, a large deletion in exon 3 [42].

To deepen the structural comparisons among the three cell systems, we had a closer look at the CTNNB1 signaling pathway. The dual function of CTNNB1, i.e., the regulation of gene transcription in the nucleus and of cell–cell adhesion through two distinct pathways, as well as its specific degradation status via the ubiquitin-proteasome system, were well-expressed in HepaRG cells (Figure 3B). In addition, the Wnt/CTNNB1 pathway and blood factors such as oxygen and metabolic hormones play central roles in the metabolic zonation of liver cells [43]. Using protein copy numbers, we checked whether the proteome of HepaRG cells could reflect a given liver lobular zone, by considering several metabolic pathways (Figure S5). For most enzymes whose abundances were measured here, a similar trend had already been described using other methods, showing for example, high glutamine and low uraemia metabolism [44,45]. HepaRG cells exhibit a phenotype that is close to that of pericentral hepatocytes, based on the expression of CYPs and urea metabolism factors. This is also true when considering the high abundance of glutamine synthase (GluL). GluL abundance was close in HepaRG cells and PHH, but it was six times lower in HepG2. Strikingly, we observed that HepaRG cells expressed all of the enzymatic machinery of periportal hepatocytes, too. This suggests that they are able to adapt their metabolism to changes in environmental conditions, such as, for example, their ability to open up the way for experimental fields for developing strategies of zonated lobule bioprinting.

Since mitochondria are essential organelles for metabolically active cells such as hepatocytes, we checked the extent to which mitochondrial proteins were present in the proteome of HepaRG cells. Despite the fact that no mitochondria enrichment steps were performed in our study, more than 880 of the proteins that we identified in the HepaRG cells are annotated as being related to the mitochondrion (Gene Ontology, see Table S5), which hints towards the good mitochondrial functionality of these cells. We validated that point by assessing HepaRG mitochondrial membrane potential using the JC-1 dye [46], which enabled us to visualize the hyperpolarization of a large number of mitochondria under basal conditions, and their expected uncoupling (i.e., depolarization) in response to FCCP, which was used to treat cells as a compensation control (Figure 3C). These data perfectly fit with what would be expected from mature differentiated hepatocytes. However, a group of mitochondrial proteins appeared to be less abundant in HepaRG cells than in PHH (Table S5B), including a series of lipid metabolism-related factors (see below), and other proteins, for example, CYP2E1 (detoxification), alanine-glyoxylate aminotransferase 2 (AGXT2; amino acid metabolism), and proteins with a redox activity, such as selenoprotein O (SELO) and its regulator, O-phosphoseryl-tRNA(Sec) selenium transferase (SEPSECS). The translocation 12/22 feature of HepaRG cells could be involved here. Indeed, the SELO gene is, for example, known to be located on chromosome 22. However, the precise consequences of translocation 12/22 on HepaRG functions remain to be elucidated.

### 3.4. Comprehensive Detoxification Function of Highly Differentiated HepaRG Cells

Detoxification is among the main functions played by liver cells. It notably involves drug-metabolizing enzymes, which are regulated by the transcription factors aryl hydrocarbon receptor (AHR), constitutive androstane receptor (CAR), pregnane X receptor (PXR), and nuclear factor erythroid 2-related factor 2 (NRF2 or NFE2L2) [47]. HepaRG cells have already been reported to express many detoxification-related enzymes and transcription factors [48,49]. Here, we compared the relative abundance of such proteins in the three cell systems.

**Figure 4:**
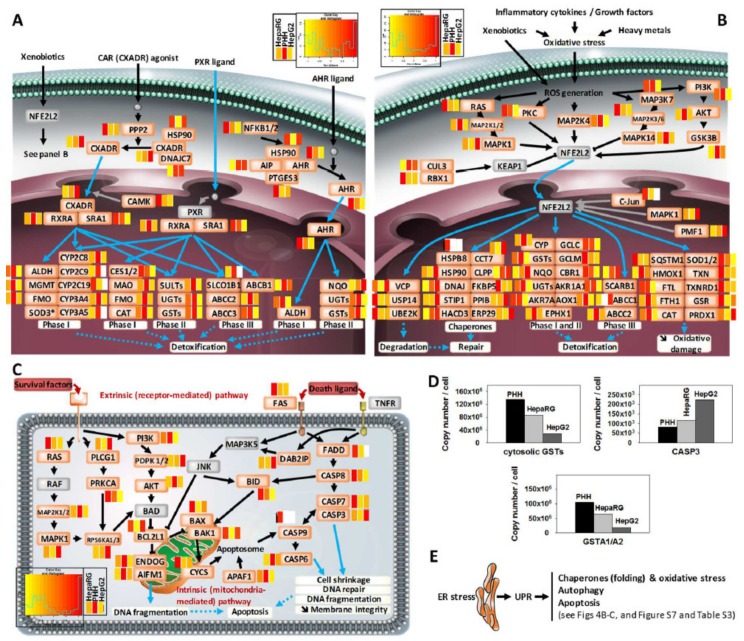
Liver-like detoxification pathways in highly differentiated HepaRG cells cultivated in defined serum-free medium (0.5% DMSO, growth factor supplementation). (**A**) Relative differences in protein abundances in HepaRG and HepG2 cells versus PHH, shown as heatmaps for xenobiotic metabolism signaling pathways (white boxes: proteins not seen). Non-detected proteins are colored in grey. See also Table S5 and Figure S6. * detected in the secretome of HepaRG cells only. (**B**) Relative differences in protein abundances in HepaRG and HepG2 cells versus PHH, shown as heatmaps for the NRF2 (NFE2L2)-mediated oxidative stress response (**C**) Relative differences in protein abundances in HepaRG and HepG2 cells versus PHH, shown as heatmaps for the apoptosis pathways. (**D**) Abundances of glutathione-S-transferases and caspases. (**E**) Proteins of the unfolded protein response (UPR) are well-expressed in HepaRG cells.

HepaRG cells were clearly highlighted as being closer to PHH than HepG2 cells (Figure 4A,B, see also Figure S6). Indeed, most of the proteins of the detoxification pathways were also detected PHH as in HepaRG, while 1/3 of them were not seen in HepG2 (see Table S5B). The latter includes Phase I (e.g., CYP2A6, CYP2C9, CYP2C19 and CYP3A5, CYP4A11, FMO3 and FMO4, and ALDH1L1 and ALDH3A1) and Phase II (e.g., SULT1B1, SULT1E1, GSTT2, and several UGTs) drug-metabolizing enzymes, as well as factors of Phase III detoxification (e.g., OATP2 or SLCO1B1 and HSP90B1 or endoplasmin) and antioxidant systems (e.g., the superoxide dismutase enzyme SOD2). In addition, the copy numbers of proteins that were commonly detected in all three cell systems were generally closer in HepaRG versus PHH than HepG2 versus PHH (Figure 4A). This is well-corroborated by previous mRNA expression assays and enzymatic measurements [50]. Nevertheless, it may be pointed out that several enzymes of the alcohol metabolism pathway were less abundant in HepaRG cells than in PHH (CYP2E1, ADH4, ADH6, ADH1C; see Table S5B). Despite the reduced levels of such enzymes, this pathway has been shown to remain highly modulatable and sensitive to various compounds in HepaRG cells [51].

Special attention was paid to the Keap1-NRF2 system, which is crucial in controlling antioxidant defenses (Figure 4B). Most of the proteins that are downstream of NRF2 transcriptional activity and responsible for cellular defense processes (i.e., degradation/repair, detoxification, reduction of oxidative damage), were detected in HepaRG cells. Their expression levels were generally closer to those in PHH, relative to the values in HepG2 cells, and approximately a fifth of these proteins were not seen in HepG2 cells (Figure 4B and Table S5B). Several proteins were three to >1000 times less abundant in HepG2 than HepaRG versus PHH cells (e.g., CYPs, UGTs, GSTs, AKR1A1, CAT). This suggests that the cytoprotective capability of the HepaRG cells may be comparably efficient to PHH, which is not the case for HepG2. Interestingly, mitochondrial superoxide dismutase (SOD2), which is key to controlling reactive oxygen species (ROS) levels, appeared to be more abundant in HepaRG cells than in PHH (roughly 8000 times). Such a high expression level in HepaRG cells may be linked to the use of the defined serum-free medium, a question to tackle in future studies. Indeed, this could enhance the detoxification response in HepaRG cells, and favor their resistance to the toxic effects of various drugs. In addition, high levels and activity of MnSOD (SOD2) in the G_0_/G_1_ cell cycle phase are believed to be critical for regulating entrance of cells into quiescence [52]. Therefore, as a survival modulator and “tumor suppressor”, SOD2 could contribute to control the prolonged survival and growth arrest that characterizes mature HepaRG hepatocytes [53]. This is also possibly the case for NAD(P)H dehydrogenase (quinone) 1 (NQO1; see Table S5 and Figure S6).

Because apoptosis is essential for the maintenance of liver homeostasis, we compared the abundance of apoptotic factors in the three cell systems (Figure 4C). Overall, HepaRG cells and PHH were quite close, while HepG2 appeared less well-equipped. For example, regarding the extrinsic (receptor-mediated) apoptotic pathway, the tumor necrosis factor receptor FAS was more abundant in HepaRG than in HepG2 cells, and FAS-associated death domain protein (FADD) was not detected in HepG2. Concerning the intrinsic (mitochondria-mediated) apoptotic pathway, the abundances of the most important apoptosis-inducing (AIFM1, BAX, BAK1, BID, APAF1) or -inhibiting (BCL2L1, BID, APAF1) factors in HepaRG were well-balanced and roughly similar to those in PHH (no more than 2–5 times lower or higher; see Table S5B). Finally, for the executing enzymes, caspase 9 (CASP9) was only detected in HepaRG cells, and the caspase 3 (CASP3) expression level was relatively close in the three cell systems. We next explored the contribution of GSTs to apoptosis regulation via caspase 3 inhibition as a functional hotspot of hepatocytes [54]. We noticed that the abundance of cytosolic glutathione-*S*-transferases (GSTT2B, GSTT1, GSTO1, GSTM4, GSTM3, GSTA1/A2) and caspase 3 (CASP3) globally followed an inverse ‘copy number gradient’ in the three cell models, suggesting that their interrelationship in HepaRG cells would better reflect the actual situation in PHH than that in the HepG2 cells (Figure 4D). Additionally, the liver-specific form of GSTA1 was the most abundant GST isoform, exhibiting levels that were 1.6- and 5.8 times lower in HepaRG and HepG2, respectively, versus PHH cells. Finally, proteins of the unfolded protein response (UPR) were well-expressed in HepaRG cells (Figure 4E), showing that these cells hold potential for studying the underlying mechanisms of endoplasmic reticulum (ER) stress, which extends the findings of our previous study [55]. For example, HSP90B1 (endoplasmin) and HSPH1 are well-detected in HepaRG, but absent from HepG2 data, and the 15 HSPs that were detected in all three cell systems displayed copy numbers that were generally closer between the HepaRG cells and PHH (see Table S5).

### 3.5. Energy Metabolic Functions of Highly Differentiated HepaRG

**Figure 5:**
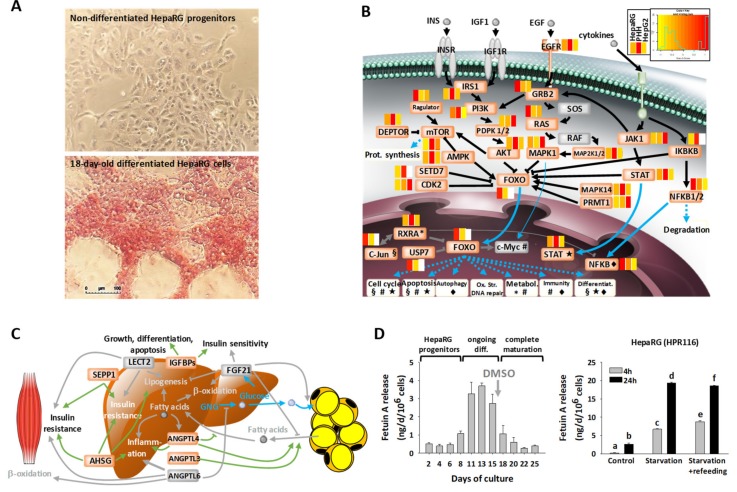
Metabolic pathways expressed by highly differentiated HepaRG cells. (**A**) Periodic acid-Schiff staining showing glycogen storage in 18-day-old differentiated HepaRG cells (differentiation medium: 1.7% DMSO, 10% FCS) but not in undifferentiated progenitor cells. (**B**) Relative differences of protein abundances in HepaRG cells (defined serum-free medium: 0.5% DMSO, growth factor supplementation) and in HepG2 cells versus PHH, shown as heatmaps for the factors of the FOXO signaling pathway (white boxes: proteins not seen). Non-detected proteins are colored in grey. See also Table S5. (**C**) Major hepatokines (in orange) identified in the secretome of HepaRG cells (defined serum-free medium). See also Table S5. (**D**) Release of Fetuin A by HepaRG cells maintained in the differentiation medium at different states of differentiation (diff.), and in response to 24 hr of fasting, and refeeding for 16 hr with palmitate (means ± SEM of three determinations). Superscript letters indicate significantly different values (analysis of variance (ANOVA) and post hoc Tukey tests; *p* < 0.05).

The liver is central to body energy metabolism, regulating, for example, glucose and lipid metabolism, storing glycogen, and synthesizing proteins. As reported above, the proteome of HepaRG cells contained a high number of carbohydrate and lipid metabolism-related proteins (Figure S2B). More precisely, HepaRG cells expressed key enzymes of glycogen metabolism (e.g., GYS, PYG, AGL), gluconeogenesis (e.g., G6Pase, PCK, Aldolase B), and glycolysis (e.g., PKLR) (Tables S5–S6 and Figure S5). From a functional point of view, we show here that differentiated HepaRG cells have the potential to store glycogen (Figure 5A). Enzymes of lipid synthesis and degradation were also well-expressed in HepaRG cells (Tables S5–S6 and Figure S5), except for few enzymes of the fatty acid oxidation pathway (e.g., SLC25A20 and ACADS), which appeared to be less abundant in HepaRG cells than in PHH (see Figure 2D and Table S5). Altogether, these results were in good agreement with previous studies that used biochemical or molecular biology methods to describe the metabolic potential of HepaRG cells [11,56,57]. Therefore, HepaRG cells may represent a good model system for studying the flexibility of glucose and fatty acid metabolism, the perturbation of which links liver zonation and metabolic liver diseases [58].

The forkhead box O (FOXO) family of transcription factors consists of essential integrators of energy metabolism. We observed that the protein components of the most important pathways interacting with FOXO, namely PI3K/AKT, RAS/RAF/MAPK, DEPTOR/mTOR, JAK/STAT, and IKBKB/NFKB, were well-detected in HepaRG cells (Figure 5B, see also Table S5). Levels of these components were generally closer between HepaRG and PHH than between HepG2 and PHH. Moreover, few proteins were detected only in HepaRG cells and PHH, including USP7 and FOXO1 (31 and 57 times more abundant in HepaRG cells, respectively). FOXO1 activity is regulated through interaction with USP7 [59], suggesting that gluconeogenic, glycolytic, and lipogenic gene expressions are tightly controlled in HepaRG cells, as is the case in hepatocytes in vivo. Similarly, the crucial role played by FOXO1 in coordinating the balance between quiescence, self-renewal, and differentiation of progenitors and mature hepatocytes, which notably involves MnSOD (SOD2, see above) in other liver cells [60], was also expected to be tightly regulated in the HepaRG cell line.

The mTOR pathway is of particular importance during cancer, obesity, and type 2 diabetes. In comparison to PHH cells, expression levels of mTOR and DEPTOR were found to be lower in both HepaRG (22 and three times, respectively) and HepG2 (two and 20 times, respectively) cells (Figure 5B and Table S5). The low abundance of mTOR in the HepaRG cells had already been shown, using antibody-based assays [61]. DEPTOR is a stemness factor that regulates pluripotency through the inhibition of mTOR signaling [62]. Hence, high DEPTOR/low mTOR abundances in HepaRG cells could help, together with the low expression of the tumor suppressor SPTBN1, mentioned above, balancing pluripotent cell renewal and differentiation in this cell system.

We also established here the first ever secretome analysis of HepaRG cells, and our data suggested good secretory activity (see Figure 2C). Apolipoproteins were remarkably well-expressed and secreted by HepaRG cells (Table S5); several isoforms could be easily detected (e.g., APOB and APOE at 0.5 and 104.1 ng/day/10^6^ cells, respectively). The same remark applied to haptoglobin, which was more abundant in HepaRG cells than PHH (700 times), and secreted at a rate of 1.8 µg/day/10^6^ cells (Table S5). Several hepatokines, which are essential to energy metabolism [63], were also well-secreted by HepaRG cells (Figure 5C). For instance, six of the seven insulin-like growth factor-binding proteins (IGFBPs) were detected, with IGFBP2 appearing as the most abundantly secreted member of this family (435 ng/day/10^6^ cells; Figure 2C and Table S5). HepaRG cells were also found to secrete the major hepatokines known to play a role in the crosstalk between the liver and muscle and adipose tissue, including selenoprotein P (SEPP1), fetuin-A (AHSG), and the angiopoietin-related proteins ANGPTL3 and ANGPTL4 (Figure 5C). We further characterized Fetuin A secretion, with rates calculated at 12 ng/day/10^6^ cells from secretome MS data (see Table S5) and measured in the same range (1–4 ng/day/10^6^ cells) using enzyme-linked immunosorbent assay (ELISA) assay (Figure 5D). In an additional experiment, we observed a drastic increase of its secretion during the differentiation process of HepaRG cells (six-fold; Figure 5D), from the progenitor state to hepatocyte differentiation. Fetuin A then stabilized at low levels when reaching complete hepatocyte maturation in the presence of DMSO. Moreover, the secretion of Fetuin A responded well to 24 hr of serum starvation (7–8-fold increase), either followed or not by refeeding with a glucose/palmitate-rich medium. This was consistent with the reported increased expression of hepatic Fetuin A, which has been reported in response to ER-stress induced by starvation and exposure to an excess of substrates, such as glucose or fatty acids [64].

### 3.6. Disease-Related Pathways in Highly Differentiated HepaRG, a Promising Field of Application for the HepaRG Cell System

The ability of hepatocyte-like cell model systems to express disease-related pathways could open up a wide field of application. Indeed, chronic liver diseases represent a major threat to public health, worldwide. Impairment of bile production and -flow causes liver cell injury that can develop into cancers, fibrosis, and cirrhosis [65]. Moreover, liver diseases are most often associated with insulin resistance and steatosis [66]. Therefore, we examined the suitability of the HepaRG cell system for studies of liver cholestasis and their ability to respond to insulin.

**Figure 6:**
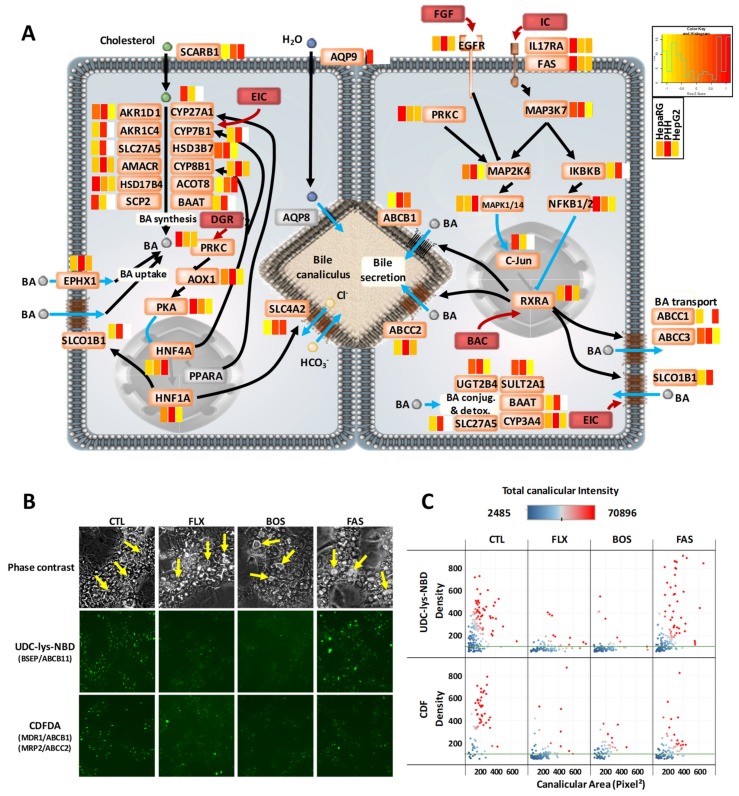
Cholestasis-related pathways in highly differentiated HepaRG cells. (**A**) Relative differences of protein abundances in HepaRG cells (defined serum-free medium: 0.5% DMSO, growth factor supplementation) and in HepG2 cells versus PHH, shown as heatmaps for bile acid (BA) metabolism (white boxes: proteins not seen). Non-detected proteins are colored in grey. See also Table S5. BAC: bile acid concentration; DGR: decreased glucagon responsiveness; EIC: estrogen-induced cholestasis, IC: inflammatory cytokines. (**B**) Imaging of bile canaliculi morphology, and bile acid trafficking and efflux in response to exposure (3 h) of HepaRG cells (differentiation medium: 1.7% DMSO, 10% FCS) to flucloxacillin (FLX), bosentan (BOS) and fasudil (FAS), compared to untreated control cells (CTL). Yellow arrows indicate bile canaliculi vesicles. UDC-lys-NBD and CDFDA: ursodeoxycholic acid and CDFDA fluorescence probes. (**C**) Image analysis from 150 bile canaliculi per condition allowed for the estimation of canalicular area (pixel²), and the total amount (intensity; arbitrary fluorescence units) and density (intensity per surface unit) of fluorescent probes transported to canaliculi. Each dot represents a canaliculus.

Highly polarized bile canaliculi structures are particularly numerous in HepaRG cells. We observed that HepaRG cells comprehensively express the molecular equipment necessary for bile acid metabolism. More precisely, abundance levels of proteins involved in bile acid synthesis, conjugation, detoxification, and transport were generally close between HepaRG and PHH, with HepG2 appearing less well-equipped (Figure 6A and Table S5). One example is aldehyde oxidase (AOX1), which was three times less abundant in HepaRG, but ~3000 times lower in HepG2 cells versus PHH. In addition, AOX1 levels appeared to be more stable in HepaRG cells than PHH. Indeed, the maximal fold change among the three batches of HepaRG cells that we compared was 1.2, while the number of AOX1 copies varied in a ratio of one to two, depending on the donor in PHH cells (see the PHH proteomics data). AOX1 is under the transcriptional control of NRF2, and it is known to be inhibited by many drugs or chemicals. Because toxic bile acids inhibit the NRF2 pathway, the resulting downregulation of AOX1 could also contribute, together with the downregulation of GSH enzymes [67], to liver injury during drug-induced cholestasis, due to ROS accumulation. In the same vein, the nuclear receptor farnesoid X receptor (FXR), which plays a key role in physiological bile acid synthesis/secretion, has already been shown to be expressed in HepaRG cells at the mRNA level [68]. This is expected to enable studies into how FXR regulation of bile acid metabolism and its possible defects are linked to cholestasis. To go into further detail, we evaluated the functionality of the bile acid transport machinery in HepaRG cells (Figure 6B). We previously demonstrated that bile acid efflux can be impaired by cholestatic drugs that induce either the constriction or dilatation of bile canaliculi, by altering ROCK/MLCK activity in HepaRG cells and PHH [37]. Here, we used phase contrast microscopy to visualize the morphology of bile canaliculi vesicles and two complementary probes to trace bile acid transport via BSEP/ABCB11 or ABCB1/ABCC2 transporters, and evaluate trafficking and efflux to bile canaliculi (Figure 6B,C). We showed that HepaRG specifically responded to exposure (3 h) to cholestatic drugs such as flucloxacillin (FLX), bosentan, and fasudil (BOS and FAS, respectively), as seen from the marked dilatation of bile canaliculi. Moreover, bile acid trafficking and efflux to bile canaliculi were clearly inhibited by FLX and BOS. An effect of FAS on bile acid trafficking was less evident, although probe accumulation in canaliculi seemed to occur. Overall, HepaRG cells appeared to be good and probably unique models for studying cholestasis-related mechanisms.

**Figure 7:**
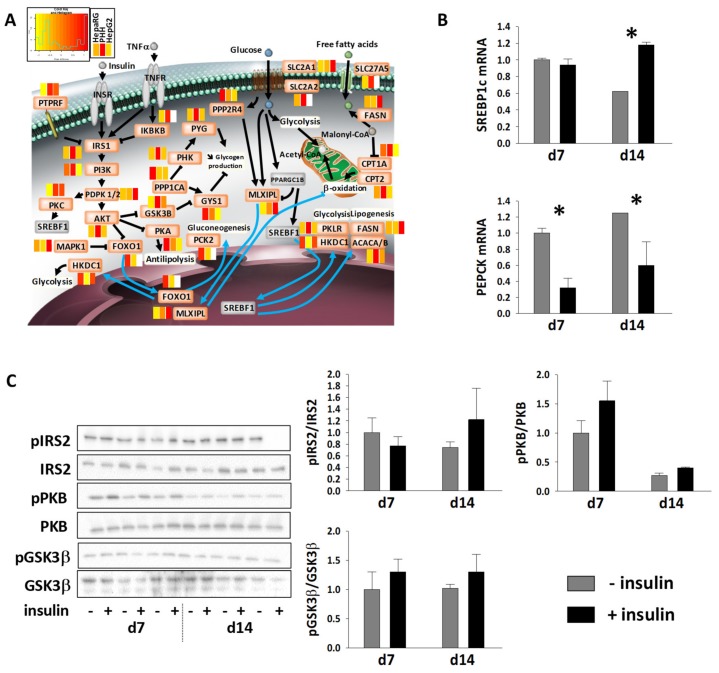
Insulin metabolism in highly differentiated HepaRG cells. (**A**) The relative difference of protein abundances in HepaRG (defined serum-free medium: 0.5% DMSO, growth factor supplementation) and in HepG2 cells versus PHH, as shown as heatmaps for factors of insulin signaling/resistance pathways (white boxes: proteins not seen). Non-detected proteins are colored in grey. See also Table S5. (**B**) mRNA levels of SREBP1c and PEPCK in differentiated HepaRG cells (differentiation medium: 1.7% DMSO, 10% FCS) in response to insulin stimulation (means ± SEM of three determinations). * indicates significant differences due to insulin stimulation for a given time point (ANOVA and post hoc Tukey tests; *p* < 0.05). (**C**) Phosphorylation status of IRS2, PKB, and GSK3β in differentiated HepaRG cells in response to insulin stimulation (means ± SEM of three determinations).

The comparison of abundances of proteins involved in insulin signaling/resistance pathways in cell lines versus PHH was at the advantage of the HepaRG cells, with a higher number of proteins being detected, and levels being generally close to those in PHH (Figure 7A). Various transporters for glucose (e.g., SLC2A1 and SLC2A2) and fatty acids (e.g., SLC27A5) were detected in HepaRG cells, while SLC2A2 and SLC27A5 were not seen in HepG2 (Table S5). This suggests that the uptake of substrates is very active in HepaRG cells. We already mentioned above that enzymes of lipogenesis and carbohydrate metabolism are also well-expressed in HepaRG cells (Figure 2D, Tables S5–S6 and Figures S2 and S5). This is in line with the fact that steatosis is induced in differentiated HepaRG cells exposed to various fatty acids [56]. Moreover, components of the insulin signaling pathway were well expressed in HepaRG cells (Figure 7A), at levels that are generally close to those in PHH (IRS1 and IRS2, PIK3R1 or PI3K, and PDPK1/2). Factors like IKBKB, FOXO1 and the limiting phosphoenolpyruvate carboxykinase enzymes (PCK2 and PCK1) were also well-detected in both HepaRG and PHH cells, while they were not present in HepG2 data. Additionally, components of the glucagon signaling pathway also appeared to be well-represented in the proteome of HepaRG cells (see Table S5), with, for example, the cyclis AMP (cAMP)-dependent protein kinase catalytic subunit beta (PRKACB) being 10 times more abundant in HepaRG than PHH cells, while not detected in HepG2. Overall, these data suggest that HepaRG cells should properly respond to insulin and glucagon. In a 6 hr insulin stimulation assay, we confirmed that the expected hepatic transcriptional response to insulin was observed for two key hepatic insulin responsive genes, namely SREBF1 (or SREBP-1c) and PCK1 (or PEPCK). Upon stimulation (Figure 7B), mRNA levels of SREBF1 and PCK1 were indeed increased and decreased, respectively, reflecting increased lipogenesis and inhibited gluconeogenesis. We thereafter validated short-term insulin response in the HepaRG model, showing that the phosphorylation status of IRS2, PKB and GSK3β tended to increase after 15 min of insulin stimulation (Figure 7C), as expected. The increase nevertheless remained moderate (<2 times), which might be linked to the fact that cells were grown in an insulin-rich medium. Insulin deprivation (overnight) prior to assays was thus not sufficient to reset basal insulin stimulation. Moreover, basal phosphorylation of PKB exhibited a decrease from day 7 to day 14, indicating that the differentiation state of HepaRG cells keeps enhancing over time. Although experimental conditions (e.g., culture medium, dose response assessment) have to be improved, HepaRG cells nevertheless appear as a very good model for studying liver disease mechanisms that are related to insulin signaling/resistance.

## 4. Conclusions

Because of their highly active hepatocyte-like metabolism, HepaRG cells have taken front stage amongst all immortalized human hepatic cell lines that have been developed to-date, notably for hepatotoxicity-related studies. Here, we show that they exhibit structural and functional aspects that are representative of hepatocytes, which extends their suitability to a wider variety of applications, e.g., for studies of energy metabolism, secretory activity, and disease-related mechanisms of hepatocytes. In particular, HepaRG cells uniquely offer the opportunity to work on the mechanisms underlying hepatic diseases such as cholestasis or insulin resistance in diabetes. In addition, proteomics data enabled us to pinpoint previously unseen characteristics, which reveal new mechanisms of interest in HepaRG cells, including i) their expected ability to be used as representative models of either pericentral or periportal hepatocytes, and ii) the functional interaction between apoptosis and glutathione-*S*-transferase regulation. Proteomics also highlighted several proteins at levels that are very different to those in PHH (CTNNB1, SOD2, SPTBN1, FOXO1, Deptor/mTOR, laminin-B1, and proteins of the cell cycle). This may be involved in HepaRG transdifferentiation properties, and the altered balance between senescence and proliferation that is generally observed in cancers. The HepaRG cell system can thus also be expected to open the way to new investigations that are related to the fields of stem-like cells and liver cancer.

## Supplementary Materials

The following are available online at https://www.mdpi.com/2073-4409/8/2/192/s1, Supplementary Materials and Methods, Figure S1: Karyotyping of HepG2 and HepaRG cells, Figure S2: Functional annotations of HepaRG intracellular and secreted proteomes, Figure S3: Master regulators controlling the hepatic phenotype of HepaRG cells, Figure S4: Comparison of intracellular proteomes of HepaRG, PHH, and HepG2 based on estimated copy numbers after LOESS normalization, Figure S5: Zonation of hepatocyte metabolism in vivo and of differentiated HepaRG cells, Figure S6: The abundance ratios of detoxification-related proteins in the three cell systems, Table S1: List of primers for RT-qPCR, and antibodies for Western-blot analyses and immunocytochemistry, Table S2: Total protein content, cell size, and intracellular protein copy numbers of PHH, HepaRG, and HepG2 cells, Table S3: Fundamental differences between HepaRG and HepG2 cells, Table S4: Proteomics data for the comparison of three different batches of cryopreserved, differentiated HepaRG cells, Table S5: Overall proteomics data in HepaRG, HepG2, and primary human hepatocytes, Table S6: 1D and 2D annotation enrichment analyses using protein-copy no ratios (log_10_) among the three cell systems, Table S7: List of the proteomic structural hotspots of HepaRG proteins; comparison with PHH and HepG2 cells. References [46,56,69] are cited in supplementary materials.
