# Supplementary material for: In-Depth Proteome Analysis Highlights HepaRG Cells as a Versatile Cell System Surrogate for Primary Human Hepatocytes
Source: Cells. 2019 Feb 21;8(2):192. doi: 10.3390/cells8020192 (PMC6406872; doi:10.3390/cells8020192)
Supplement: Supplementary file 1 [file cells-08-00192-s001.zip › cells-417497-supplementary_proofread.docx]

Supplementary Materials and Methods

**In-Depth Proteome Analysis Highlights HepaRG Cells as a Versatile Cell System Surrogate for Primary Human Hepatocytes**

**Georg Tascher ^1,2^, Audrey Burban ^3^, Sandrine Camus ^4^, Marine Plumel ^1^, Stéphanie Chanon ^5^, Remy Le Guevel ^6^, Valery Shevchenko ^4^, Alain Van Dorsselaer ^1^, Etienne Lefai ^5^, Christiane Guguen-Guillouzo ^3,4^ and Fabrice Bertile ^1,^***

^1^ Laboratoire de Spectrométrie de Masse BioOrganique, CNRS, IPHC UMR 7178, Université de Strasbourg, F-67087 Strasbourg, France; marine.plumel@gmail.com (M.P.), vandors@unistra.fr (A.V.D.)

^2^ Institute of Biochemistry II, Goethe University Hospital, D-60590 Frankfurt am Main, Germany; tascher@med.uni-frankfurt.de

^3^ INSERM U1241 NuMeCan, Université de Rennes 1, F-35033 Rennes, France; burban.au@gmail.com (A.B.), christiane.guillouzo@univ-rennes1.fr (C.G.G.)

^4^ Biopredic International, Parc d’Affaires de la Bretêche, F-35760 St Grégoire, France; sandrine.camus@biopredic.com (S.C.), valery.shevchenko@biopredic.com (V.S.)

^5^ CarMeN Laboratory, INSERM, INRA, University of Lyon, F-69310 Pierre-Bénite, France; stephanie.chanon@univ-lyon1.fr (S.C.), etienne.lefai@inra.fr (E.L.)

^6^ ImPACcell platform, Biosit, Université de Rennes 1, F-35043 Rennes, France; remy.leguevel@univ-rennes1.fr

***** Correspondence: fbertile@unistra.fr; Tel.: +33-368-852-681

Received: 14 December 2018; Accepted: 18 February 2019; Published: date

1. Mitochondrial Activity in HepaRG Cells

The red–orange membrane-permeant JC-1 dye (5,5’,6,6’-tetrachloro-1,1’,3,3’-tetraethylbenzimidazolo carbocyanine iodide), when positively-charged, readily accumulates in active mitochondria, due to their relative negative charge. On the reverse, the membrane potential of depolarized or inactive mitochondria is decreased and they fail to sequester JC-1. This dye therefore enables one to assess the mitochondrial permeability transition (MPT) [1].

JC-1 (#T3168; Invitrogen, ThermoFisher Scientific, Rockford, IL, USA) was used at a concentration of 7 µM corresponding to the maximum solubility value. Six days after seeding, HepaRG cells (HPR116 cultivated in the differentiation medium) were exposed to the medium containing JC-1 over two successive exposures, as described by Pernelle et al. [1]. The specificity of JC-1 for mitochondria was assessed by using the uncoupling agent FCCP (phenylhydrazone; #C2920; Sigma Aldrich, St. Louis, MO, USA) added at a concentration of 100 µM, to the culture 15 min before the two-charge JC-1 protocol.

2. Glycogen Storage Assay in HepaRG Cells

Accumulation of glycogen in proliferating HepaRG progenitors and 18-day-old cultures (HPR116 in the differentiation medium) was assessed by using the periodic acid–Schiff (PAS) assay, following the manufacturer’ recommendations (#395B; Sigma Aldrich, St. Louis, MO, USA).

3. Fetuin A Secretion from HepaRG Cells

Six days after seeding, fetuin A secretion from differentiated HepaRG cells (HPR116 maintained in the differentiation medium) was measured by collecting the medium from HepaRG cells at different time points during the progression of hepatocyte differentiation (N = 3/time point), from progenitor stage to maturation, using the human fetuin A ELISA kit (#DFTA00; R&D Systems Europe, Lille, France). The influence of starvation followed or not by refeeding (N = 3/condition) on fetuin A secretion was also tested. Differentiated cells (HPR116; 6 days after seeding) were washed three times and incubated for either 4 hr or 24 hr in the starvation medium deprived of serum, DMSO, and insulin. They were refed for 16 h with a medium containing 10% FCS, 1.7% DMSO, 2.5 µg/mL insulin, and either 5 g/L of glucose or 100 µM of palmitate according to Madec et al. [2]. Data are expressed as fetuin A release into the medium in ng/day/10^6^ cells.

4. Evaluation of Bile Acid Transport and Trafficking in HepaRG Cells

Highly polarized differentiated HPR116 cells were used at day 10 of culture in the differentiation medium (1.7% DMSO, 10% FCS). They were washed three times with an assay buffer (William’s E medium deprived of serum and DMSO, and supplemented with 0.1% bovine serum albumin (Sigma Aldrich, St. Louis, MO, USA). After the last washing step, which was performed at 37 °C for 15 min, cells were exposed for 3 hr to three referent cholestatic drugs, namely flucloxacillin, Bosentan, and Fasudil, used at a final concentration of 1.5 mM, 100 µM and 50 µM, respectively. Phase-contrast microscopy allowed for the alteration of bile canaliculi morphology (dilatation) to be visualized. To evaluate bile acid trafficking and efflux to the bile canaliculi, two different probes were prepared in the assay buffer. The CDFDA probe (5(6)-carboxy-2’,7’-dichlorofluorescein diacetate; #21884; diluted 1/200; Sigma Aldrich, St. Louis, MO, USA) was added directly to the medium at a final concentration of 3 µM, 30 min before ending drug exposure. The UDC-lys-NBD probe (3α-hydroxy-7-nitrobenzoxadiazolyl-ursodeoxycholic acid; synthesized by ICOA-University of Orléans, France) was added prior to drug exposure at a final concentration of 5 µM, and then it was discarded and replaced without washing with drug solutions. At the end of the incubations, cells were extensively washed (five times) and imaged at 490/520 nm, using the ArrayScan VTI HCS reader (Cellomics, ThermoFisher Scientific, Rockford, IL, USA) equipped with a Photometrics X1 CCD camera boasting a 14-bit dynamic range (20X objective, LD Plan Neofluar 20×/0.4 korr; Carl Zeiss, Birmingham West Midlands, UK). Image analysis of canaliculi fluorescence using HCS Studio Cell analysis software v2.0 (ThermoFisher Scientific, Rockford, IL, USA) was optimized for spot analysis (background removal, object segmentation and detection). Hence, we estimated the area (pixel²) of 150 bile canaliculi per condition, and the total amount (intensity; arbitrary fluorescence units) and density (intensity per surface unit) of UDC-lys-NBD and CDF probes transported into these canaliculi.

5. Evaluation of the Response of HepaRG Cells to Insulin

HepaRG cells (HPR116) were cultivated as described above. At day 7 and day 14 of differentiation, cells were washed twice with PBS, and then incubated overnight in the culture medium without insulin. Afterwards, they were incubated in absence or presence of human recombinant insulin (Sigma Aldrich, St. Louis, MO, USA) at a final concentration of 100 nM, either for six hours prior to PCR assays, or for 15 minutes prior to Western Blot analyses (N = 3/condition).

Total RNA was isolated by using the TRIzol reagent (Invitrogen, Courtaboeuf, France) according to the manufacturer’s instructions. First-strand cDNAs were synthesized from 1 µg of total RNAs using PrimeScript RT kit (Ozyme, Saint-Quentin-en-Yvelines, France) with a mixture of random hexamers and oligo(dT) primers, and treated with 60 units of RnaseH (Ozyme, St-Quentin-en-Yvelines, France) Real-time PCR assays were performed with a Rotor-Gene 6000 (Qiagen, Courtaboeuf, France). Different primers were used for SREBP1c and PEPCK (see Table S1), and the results were normalized by using TBP (TATA box binding protein) mRNA concentrations, measured as a reference gene in each sample.

For protein extraction, cells were then scraped into 500 μL of ice-cold lysis buffer (Tris-HCl 20 mM, NaCl 138 mM, KCl 2.7 mM, MgCl_2_ 1 mM, glycerol 5%, NP 40 1%, EDTA 5mM, Na_3_VO_4_ 1 mM, NaF 20 mM, and DTT 1 mM). Protease inhibitor cocktail (Sigma) was added extemporaneously. Protein concentration was determined by Bradford quantification. Western blotting was performed as previously described [3], with 20 µg for protein loading. All membranes were blocked with 4% BSA (bovine serum albumin, Euromedex, Souffelweyersheim, France) and probed with primary then secondary HRP antibodies (see Table S1). Chemiluminescence signals were recorded using a Chemidoc camera (Bio-Rad, Hercules, CA, USA).

References

1. Pernelle, K.; Le Guevel, R.; Glaise, D.; Stasio, C. G.; Le Charpentier, T.; Bouaita, B.; Corlu, A.; Guguen-Guillouzo, C. Automated detection of hepatotoxic compounds in human hepatocytes using HepaRG cells and image-based analysis of mitochondrial dysfunction with JC-1 dye. *Toxicol. Appl. Pharmacol*. **2011**, *254*, 256–66.
2. Madec, S.; Cerec, V.; Plee-Gautier, E.; Antoun, J.; Glaise, D.; Salaun, J.P.; Guguen-Guillouzo, C.; Corlu, A. CYP4F3B expression is associated with differentiation of HepaRG human hepatocytes and unaffected by fatty acid overload. *Drug Metab. Dispos.* **2011**, *39*, 1987–96.
3. Dessalle, K.; Euthine, V.; Chanon, S.; Delarichaudy, J.; Fujii, I.; Rome, S.; Vidal, H.; Nemoz, G.; Simon, C.; Lefai, E. SREBP-1 transcription factors regulate skeletal muscle cell size by controlling protein synthesis through myogenic regulatory factors. *PLoS One* **2012**, *7*, e50878.
