# Supplementary material for: In-Depth Proteome Analysis Highlights HepaRG Cells as a Versatile Cell System Surrogate for Primary Human Hepatocytes
Source: Cells. 2019 Feb 21;8(2):192. doi: 10.3390/cells8020192 (PMC6406872; doi:10.3390/cells8020192)
Supplement: Supplementary file 1 [file cells-08-00192-s001.zip › Figure S1_revised2.pdf]

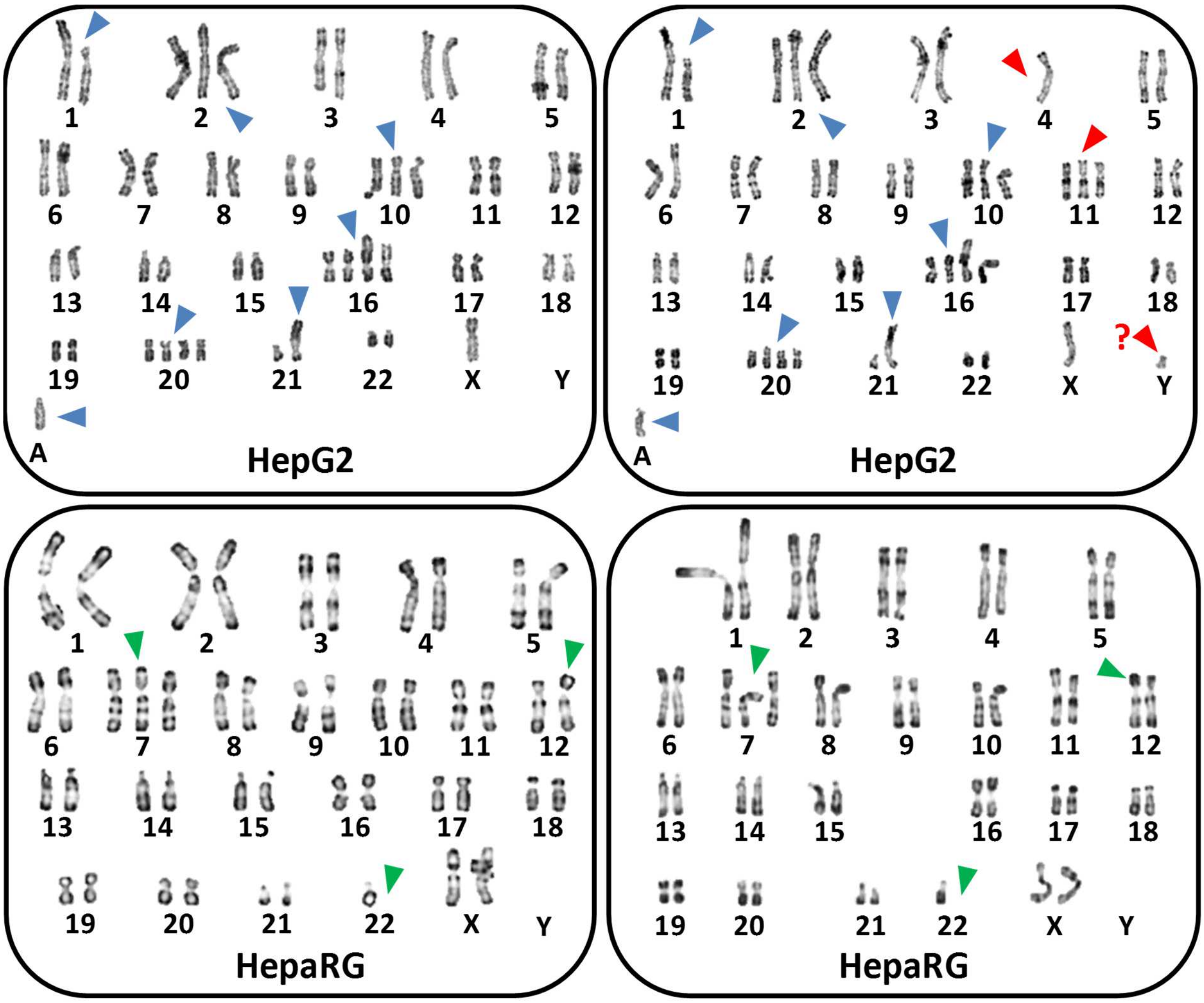

Figure S1. Karyotyping of HepG2 and HepaRG cells.
Note the high number of trisomies and abnormal chromosomes commonly observed in all HepG2 cells (blue), and unstable profiling illustrated by strong variations occurring from one cell to the other within the same HepG2 culture population (differences between left and right panels in red). On the reverse, HepaRG cells exhibit limited and constant chromosomal alterations, with trisomy 7 and translocation between chromosomes 12 and 22 (green), even in cells from different culture passages (passage 12 on the left and 16 on the right).
Interestingly, the difference in gene doses due to the abnormal karyotypes was reflected in the proteome data, with proteins encoded on Chromosomes 20 and 7 being present in significantly higher copy numbers in HepG2 (FDR < 0.05%) and HepaRG (FDR < 0.2%) cells, respectively, according to 1D annotation enrichment analysis when compared to PHH (See Table S6).
The fundamental difference in the expansion strategy of HepaRG and HepG2 cells since the cells were isolated from the original tumors and the cell lines were established is presented in Table S3.
