# Supplementary material for: In-Depth Proteome Analysis Highlights HepaRG Cells as a Versatile Cell System Surrogate for Primary Human Hepatocytes
Source: Cells. 2019 Feb 21;8(2):192. doi: 10.3390/cells8020192 (PMC6406872; doi:10.3390/cells8020192)
Supplement: Supplementary file 1 [file cells-08-00192-s001.zip › Figure S2.pdf]

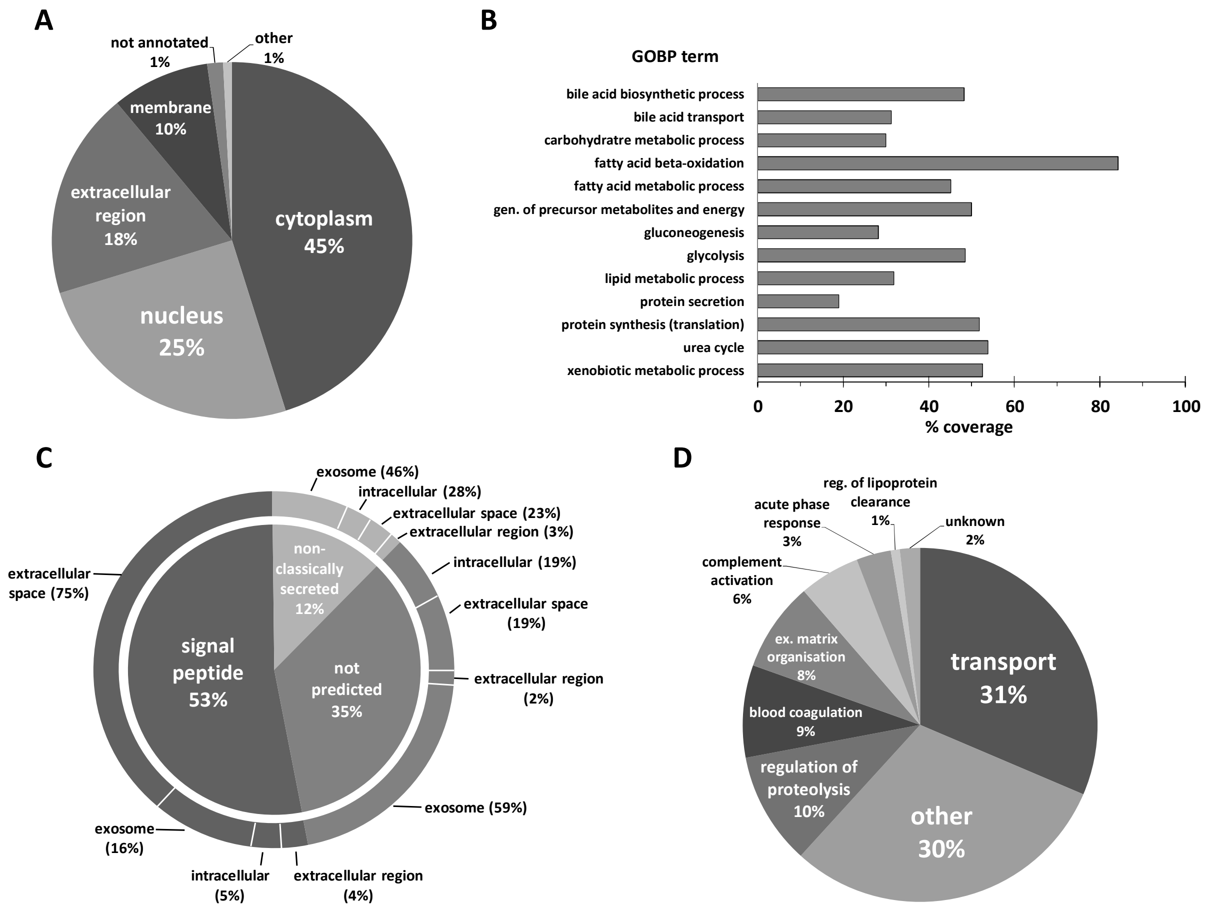

Figure S2: Functional annotations of HepaRG intracellular and secreted proteomes.
4394 intracellular proteins were identified in HepaRG cells, of which the subcellular localisation (GOCC, panel A) and coverage of assigned biological processes (GOBP, panel B) related to liver function (expressed as percentage of the total number of proteins annotated to the respective term in the Gene Ontology database) are shown. Secretion status of extracellular proteins based on the prediction of a signal peptide (SignalP) or sequence features reflecting a non-classical secretion (SecretomeP) and the corresponding localisation (GOCC) in each category (circle around the pie-chart, panel C). Biological processes (GOBP) of 272 bona-fide secreted proteins, i.e. excluding “intracellular” proteins as denoted in panel C. Note that percentages are calculated from total annotations, i.e. proteins can be attributed to more than one category (Panel D).
