# Supplementary material for: In-Depth Proteome Analysis Highlights HepaRG Cells as a Versatile Cell System Surrogate for Primary Human Hepatocytes
Source: Cells. 2019 Feb 21;8(2):192. doi: 10.3390/cells8020192 (PMC6406872; doi:10.3390/cells8020192)
Supplement: Supplementary file 1 [file cells-08-00192-s001.zip › Figure S3.pdf]

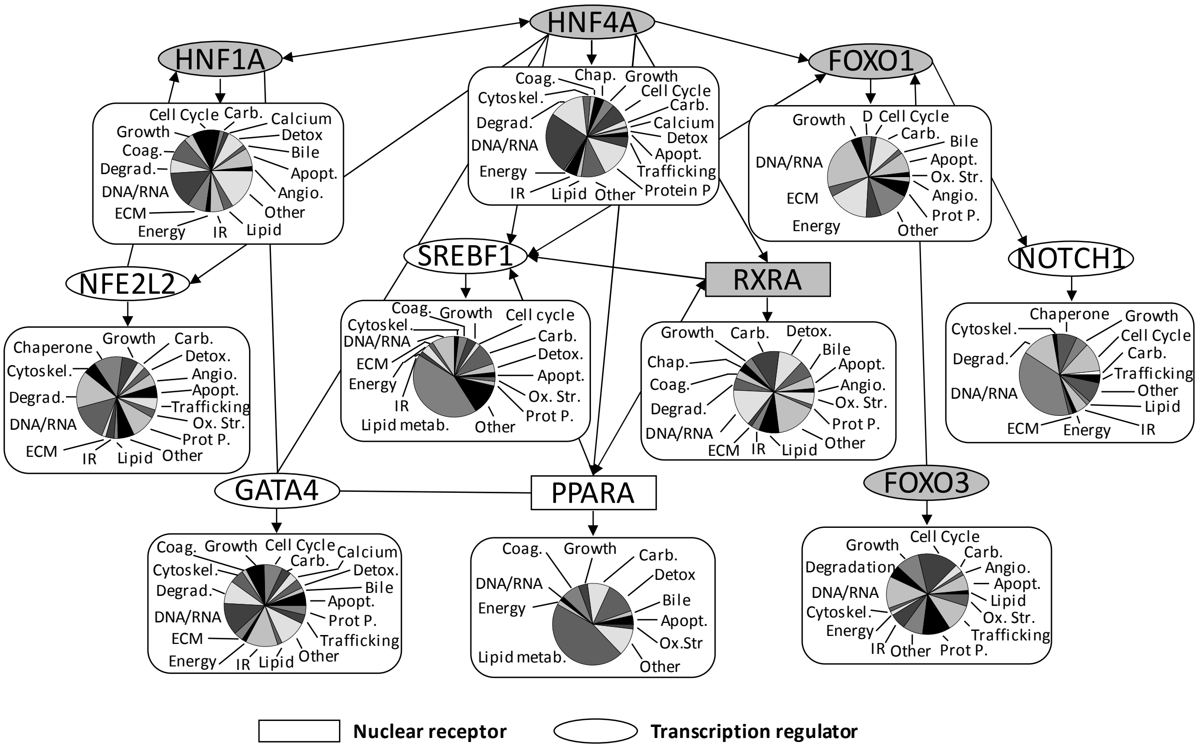

Figure S3. Master regulators controlling the hepatic phenotype of HepaRG cells.
The hepatic phenotype is controlled by several master regulators, including nuclear receptors and transcription regulators, that were detected (in grey) in HepaRG cells. Top 100 (HNF4A) or Top 50 (HNF1A, NFE2L2, SREBF1, RXRA, FOXO1, FOXO3, NOTCH1, PPRA, GATA4) master regulator-related factors detected in HepaRG cells were extracted using Ingenuity® Pathway Analysis software and were categorized using automatic extraction of corresponding KEGG and Gene Ontology annotations, whose proportion is illustrated as pie charts. Angio: Angiogenesis; Apopt.: Apoptosis; Bile: Bile secretion; Carb.: Carb. Metabolism; D: Degradation; Diff: Differentiation; DNA/RNA: DNA & RNA processing; ECM: ExtraCellular Matrix; Energy: Energy metabolism; IR: Immune Response; Ox. Str.: Resp. to oxidative Stress; Prot P.: Protein Processing.
