# Supplementary material for: In-Depth Proteome Analysis Highlights HepaRG Cells as a Versatile Cell System Surrogate for Primary Human Hepatocytes
Source: Cells. 2019 Feb 21;8(2):192. doi: 10.3390/cells8020192 (PMC6406872; doi:10.3390/cells8020192)
Supplement: Supplementary file 1 [file cells-08-00192-s001.zip › Figure S4_revised2.pdf]

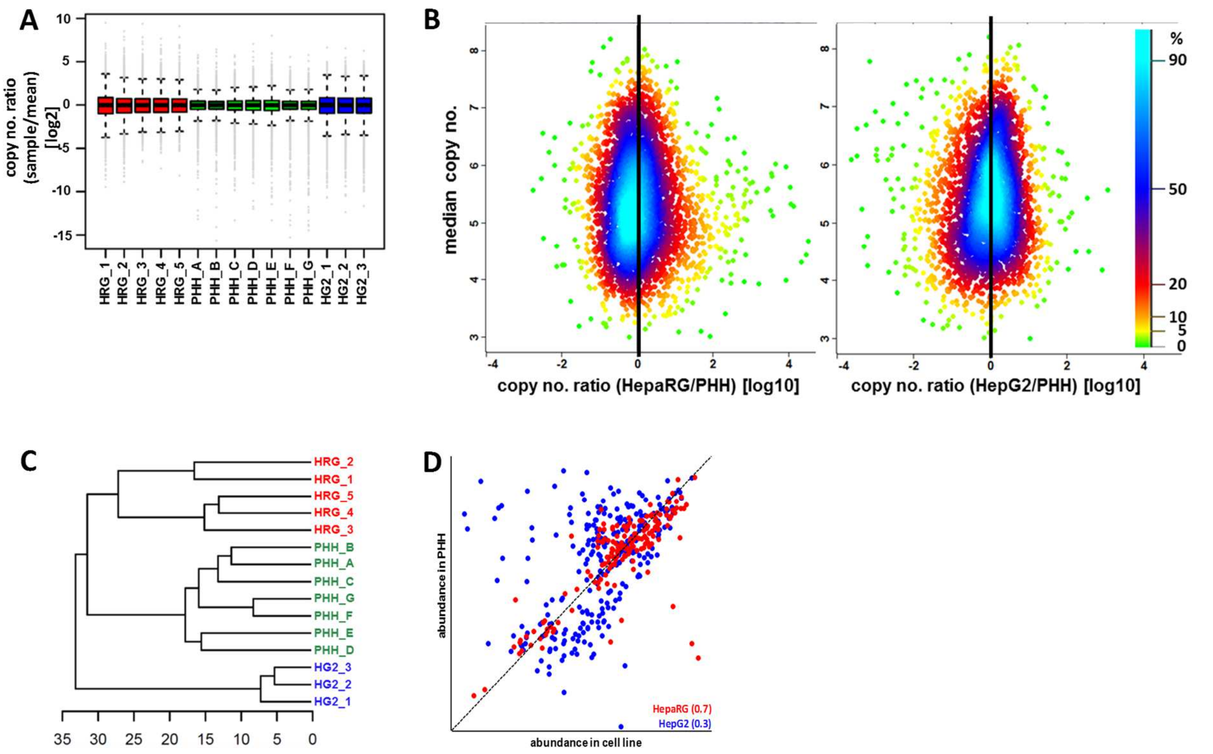

Figure S4: Comparison of intracellular proteomes of HepaRG, PHH and HepG2 based on estimated copy numbers after LOESS normalisation.

Relative log expression plot (panel A) of all samples created by the R-package "Normalyzer". Copy numbers were calculated with the "total protein approach" using total protein content and cell volumes as determined in this study. Panel B shows a scatter plot of protein copy number ratios (log10) between HepaRG and PHH (left) and HepG2 and PHH (right). Scatter plots are coloured according to the excluded fraction of data points (in %) as shown in the legend (lower right). Hierarchical clustering (panel C) was performed using only proteins without missing values in any of the samples (1630 proteins) after centering and scaling of the data. Panel D shows a scatter plot of 2D annotation enrichment scores performed on protein abundances (copy numbers) in the cell lines (HepaRG in red, HepG2 in blue) versus PHH comparing their proteome composition based on protein abundances related to functional annotation terms. In the lower right of panel D, correlation coefficients are shown.
