# Supplementary material for: In-Depth Proteome Analysis Highlights HepaRG Cells as a Versatile Cell System Surrogate for Primary Human Hepatocytes
Source: Cells. 2019 Feb 21;8(2):192. doi: 10.3390/cells8020192 (PMC6406872; doi:10.3390/cells8020192)
Supplement: Supplementary file 1 [file cells-08-00192-s001.zip › Figure S5_revised.pdf]

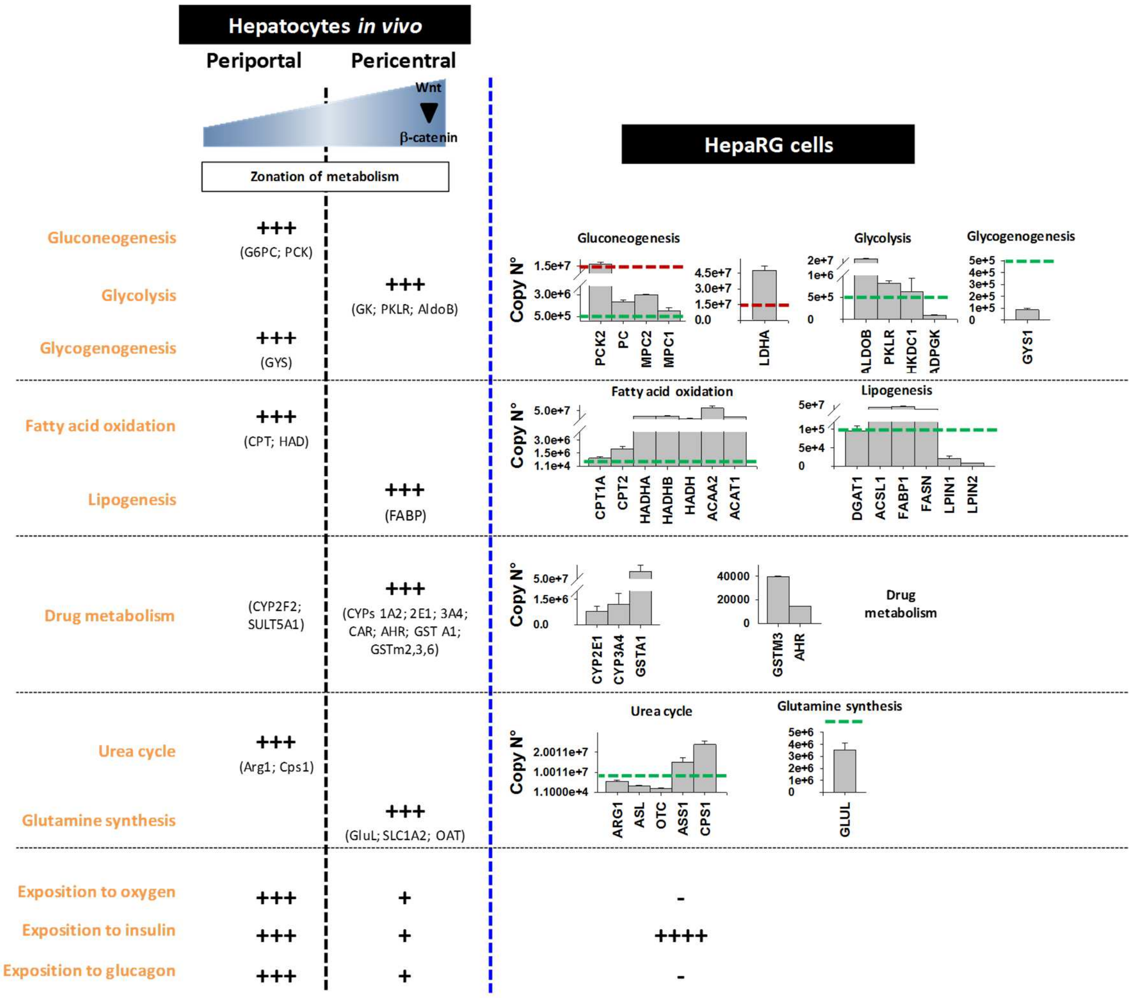

Figure S5. Zonation of hepatocyte metabolism *in vivo* and of differentiated HepaRG cells.
From literature mining, it is clear that metabolic zonation of hepatocytes *in vivo* corresponds to the functional organization of the liver lobule in which the Wnt/beta-catenin pathway and blood factors such as oxygen and hormones play central roles. We consider here the main features of the periportal zone, which is around the portal triads and the pericentral zone, which is around the central vein. HepaRG were cultivated in a defined serum-free medium (0.5% DMSO, growth factor supplementation. Using our HepaRG proteomics dataset (copy numbers of analyzed proteins), we show that although these cells are reported to exhibit a phenotype close to that of pericentral hepatocytes, they express all the enzymatic machinery of also periportal hepatocytes. In given broad metabolic functional categories, red ($1.5x10^7$ protein copies) and green ($5x10^5$ protein copies) dotted lines may be used to better appreciate differences in the levels of proteins from different pathways.
