# Supplementary material for: In-Depth Proteome Analysis Highlights HepaRG Cells as a Versatile Cell System Surrogate for Primary Human Hepatocytes
Source: Cells. 2019 Feb 21;8(2):192. doi: 10.3390/cells8020192 (PMC6406872; doi:10.3390/cells8020192)
Supplement: Supplementary file 1 [file cells-08-00192-s001.zip › Figure S6.pdf]

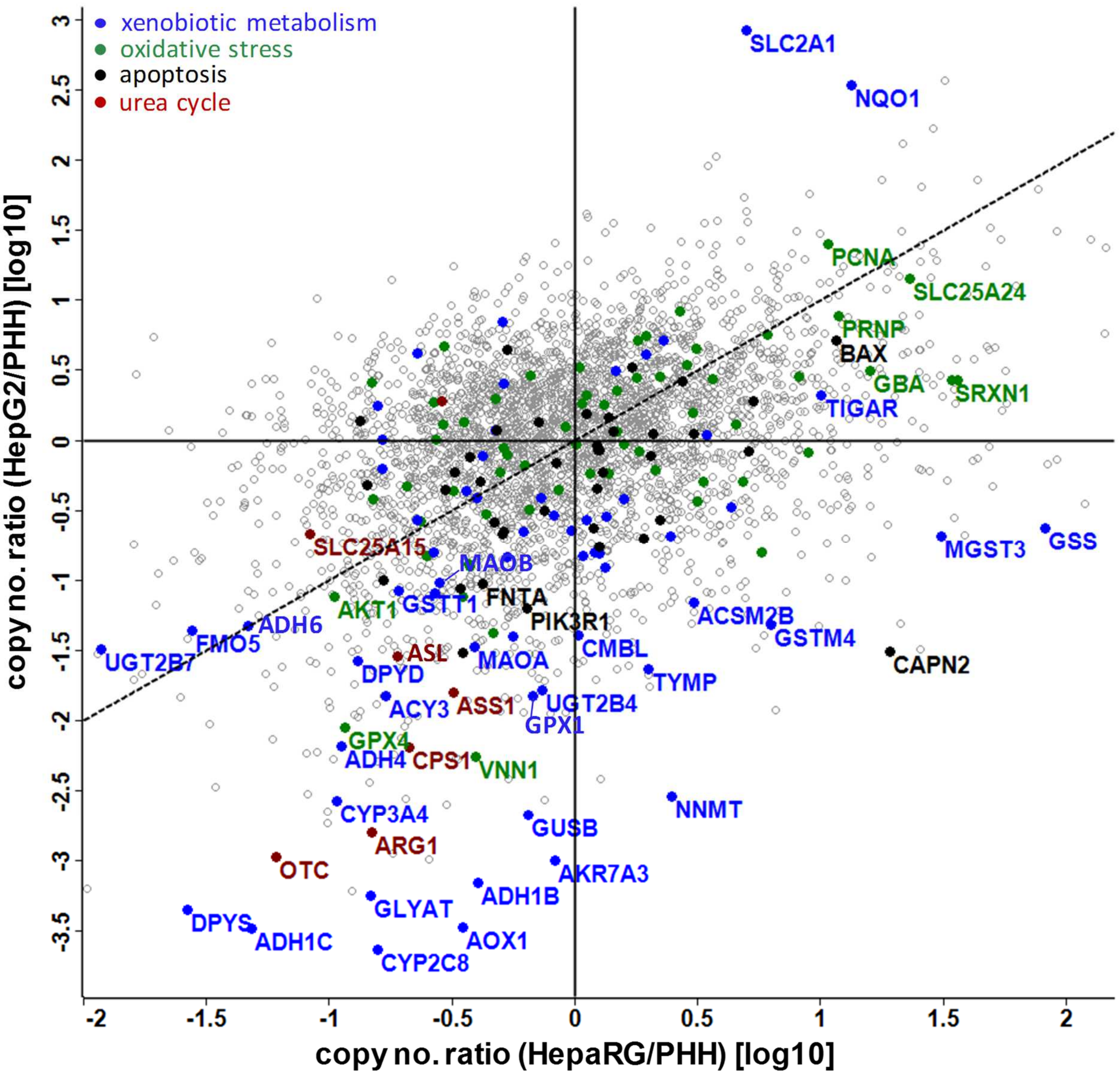

Figure S6: Abundance ratio of detoxification-related proteins in the three cell systems.
Based on estimated copy numbers (see text), the detoxification-related proteome of HepaRG cells was compared to that of PHH and HepG2 cells.
