# Supplementary material for: In-Depth Proteome Analysis Highlights HepaRG Cells as a Versatile Cell System Surrogate for Primary Human Hepatocytes
Source: Cells. 2019 Feb 21;8(2):192. doi: 10.3390/cells8020192 (PMC6406872; doi:10.3390/cells8020192)
Supplement: Supplementary file 1 [file cells-08-00192-s001.zip › Table S1_revised2.pdf]

Table S1. List of primers for RTqPCR and antibodies for Western-blot analyses and immunocytochemistry

**List of primers used for RT-qPCR**				
**Gene**		**Primer**		
SREBP1c	Forward	5'-GCAGATCGCGGAGCCATGGATTGC-3'		
	Reverse	5'-GAGGTGGAGACAAGCTGCCTGG-3'		
PEPCK	Forward	5'-GCTCTGAGGAGGAGAATGG-3'		
	Reverse	5'-TGCTCTTGGGTGACGATAAC-3'		
TBP	Forward	5′- AGACCATTGCACTTCGTGCC-3′		
	Reverse	5′-CCTGTGCACACCATTTTCCC-3′		
**List of antibodies used for Western-blot analysis**				
**Name**	**Supplier**	**Reference**	**Dilution**	**Species**
IRS2	Millipore	#06-506	1/1000	Rabbit
phospho-IRS2 [Ser731]	Abcam	#ab3690	1/1000	Rabbit
GSK3 beta	Cell Signaling	#CS9315	1/2000	Rabbit
phospho-GSK3 beta [Tyr216]	Abcam	#ab75745	1/2000	Rabbit
PKB	Cell Signaling	#4691	1/1000	Rabbit
phospho-PKB [Ser473]	Cell Signaling	#4060	1/1000	Rabbit
Anti-rabbit IgG [H+L]-HRP conjugate	Bio-Rad	172-1019	1/10000	Goat
**List of antibodies used for immunocytochemistry**				
**Name**	**Supplier**	**Reference**	**Dilution**	**Species**
ZO-1	BD Bioscience	610966	1/500	Mouse
HNF4	Abcam	AB41898	1/2000	Mouse
β-catenin	Cell Signaling	9587	1/500	Rabbit
Anti-mouse IgG [H+L] Alexa fluor 488	Invitrogen	A-10680	1/500	Goat
Anti-rabbit IgG [H+L] Alexa fluor 647	Invitrogen	A-21245	1/500	Goat
