# Supplementary material for: In-Depth Proteome Analysis Highlights HepaRG Cells as a Versatile Cell System Surrogate for Primary Human Hepatocytes
Source: Cells. 2019 Feb 21;8(2):192. doi: 10.3390/cells8020192 (PMC6406872; doi:10.3390/cells8020192)
Supplement: Supplementary file 1 [file cells-08-00192-s001.zip › Table S2_revised2.pdf]

Table S2. Total protein content, cell size and intracellular protein copy numbers of PHH, HepaRG, and HepG2 cells.

cell system	total protein/cell [pg][a]	cell size [pl][b]	intracellular proteins [g/l][b]	total protein copies/cell[b]
**PHH**	920 ± 59	6.4	144 ± 17	$1.3x10^{10}$ ± $1.3x10^{8}$
**HepaRG**	678 ± 62	2.6	264 ± 38	$1.1x10^{10}$ ± $5.0x10^{8}$
**HepG2**	420 ± 7	1.2	365 ± 42	$0.6x10^{10}$ ± $2.3x10^{7}$

[a] experimentally measured in this study, [b] calculated using experimental measurments from this study.
