# Supplementary material for: In-Depth Proteome Analysis Highlights HepaRG Cells as a Versatile Cell System Surrogate for Primary Human Hepatocytes
Source: Cells. 2019 Feb 21;8(2):192. doi: 10.3390/cells8020192 (PMC6406872; doi:10.3390/cells8020192)
Supplement: Supplementary file 1 [file cells-08-00192-s001.zip › Table S3_revised2.pdf]

	HepaRG cells	HepG2 cells
**Cell origin**	Hepatocholangiocarcinoma from adult Caucasian female	Hepatoblastoma from 15 year-old Caucasian male
**Year of cell isolation**	1999	1976
**Cell bank setting**	INSERM/Biopredic Int. 2000	ATCC bank 1980
**Passage number for cell banking**	Isolated cells from original tumor<br>Multiple frozen vials available from cultures passages 3- 4, passages 7-8, passage 9, and passage 11	Multiple passages > 100<br>Number unknown
**First description of the line**	Gripon et al. 2002[1]	Aden et al. 1979[2]
**Patent**	2004 (Passage 10)	1980 (Passage unknown)
**First karyotyping**	**2002**<br>Gripon et al. 2002[1]<br>-From INSERM/Biopredic Int. cell bank<br>-Passage 8<br>-Pseudodiploid with 46 Ch + XX<br>-Trisomy Ch 7<br>-Translocation Ch 12/22	**1980**<br>Knowles et al. 1980[3]<br>-Numerous passages<br>-Number undefined<br>-Ch1 rearrangement
**Latest Karyotyping and gene expression profiling**		**2000**<br>Wong et al. 2000[4]<br>-From ATCC but passage undefined<br>-Pseudotetraploid with 52-78 ch. + XY<br>-Trisomies of Ch 2, 14 and 20<br>Breakpoints in different Ch
	**2010**<br>Hart et al. 2010[5]<br>-From INSERM/Biopredic Int. cell bank<br>-Passage 14 or 16<br>-Gene expression levels in accordance with Trisomy Ch 7	**2009**<br>Lopez-Terrada et al. 2009[6]<br>-Numerous passages, number undefined<br>-Translocation Ch 1 / 4<br>-Trisomies 2, 16 and 20<br>-Segmental gains for Ch 6, 14, 17<br>-Large deletion in exon 3 of β-catenin
	**2016**<br>-From INSERM/Biopredic Int. cell bank<br>-Pseudodiploid<br>-Passages 11, 14 and 18<br>-Constant profiling with trisomy of Ch 7 and translocation 12/22<br>-Passage > 20 occurrence of additional trisomies (mainly Ch 8)	**2016**<br>-From ATCCR<br>-Passage undefined<br>-Variable Ch. profiling from one cell to the other<br>-Trisomies of Ch 2, 10, 11, 16, 20<br>-Rearrangements of Ch 1, 4, 21, Y

Table S3. Fundamental differences between HepaRG and HepG2 cells

This table summarizes the fundamental difference in the expansion strategy of HepaRG and HepG2 cells, since they were isolated from the original tumors and the lines were established. Owing to the bank settled with cells frozen at very early passages (only few population doublings), characteristics of HepaRG cells remain highly stable and close to the initial profiling of the line described in 2002. Indeed, we experimentally know that beyond passages 18-20, HepaRG cells become unstable transformed cells and loose many liver-specific functions. Regarding HepG2, attention was never really paid to the passage number (population doubling). At their origin, as described by Aden et al. in 1979, the cells appeared higher differentiated than those distributed today, which show numerous variable chromosomal abnormalities.

[1] Gripon, P.; Rumin, S.; Urban, S.; Le Seyec, J.; Glaise, D.; Cannie, I.; Guyomard, C.; Lucas, J.; Trepo, C.; Guguen-Guillouzo, C. Infection of a human hepatoma cell line by hepatitis B virus. *Proc Natl Acad Sci U S A* **2002**, *99*, 15655-60.

[2] Aden, D. P.; Fogel, A.; Plotkin, S.; Damjanov, I.; Knowles, B. B. Controlled synthesis of HBsAg in a differentiated human liver carcinoma-derived cell line. *Nature* **1979**, *282*, 615-6.
[3] Knowles, B. B.; Howe, C. C.; Aden, D. P. Human hepatocellular carcinoma cell lines secrete the major plasma proteins and hepatitis B surface antigen. *Science* **1980**, *209*, 497-9.
[4] Wong, N.; Lai, P.; Pang, E.; Leung, T. W. T.; Lau, J. W. Y.; Johnson, P. J. A comprehensive karyotypic study on human hepatocellular carcinoma by spectral karyotyping. *Hepatology* **2000**, *32*, 1060-1068.
[5] Hart, S. N.; Li, Y.; Nakamoto, K.; Subileau, E. A.; Steen, D.; Zhong, X. B. A comparison of whole genome gene expression profiles of HepaRG cells and HepG2 cells to primary human hepatocytes and human liver tissues. *Drug Metab Dispos* **2010**, *38*, 988-94.
[6] Lopez-Terrada, D.; Cheung, S. W.; Finegold, M. J.; Knowles, B. B. Hep G2 is a hepatoblastoma-derived cell line. *Hum Pathol* **2009**, *40*, 1512-5.
