# Supplementary material for: In-Depth Proteome Analysis Highlights HepaRG Cells as a Versatile Cell System Surrogate for Primary Human Hepatocytes
Source: Cells. 2019 Feb 21;8(2):192. doi: 10.3390/cells8020192 (PMC6406872; doi:10.3390/cells8020192)
Supplement: Supplementary file 1 [file cells-08-00192-s001.zip › Table S7_revised2.pdf]

Table S7. List of proteomic structural hotspots of HepaRG proteins, comparison with PHH and HepG2 cells.

Family or system	N	Protein Identifiers	Fold change (LOG10) of copy numbers in HepaRG or HepG2 cells vs. PHH
**Extracellular matrix**			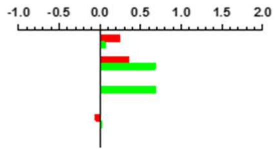
**Integrin and integrin-associated**	10	ILK, ILKAP, ITGA1, ITGA2#, ITGA3*#, ITGA5*, ITGA6, ITGAV, ITGB1, ITGB5	
**Collagen and collagen-associated**	9	COL18A1, COL4A3BP, COL7A1*#, COL8A1*#, COLGALT1, PLOD1, PLOD2, PLOD3, PLOLCE*#	
**Fibronectin, laminin, proteoglycan**	7	FN1, GPC1, GPC6, HSPG2, LAMB1, LAMC1, SDC1	
**Proteinase-associated**	16	ADAM9, ADAM15*#, SERPINA1, SERPINA3, SERPINB1, SERPINB2*, SERPINB6, SERPINB8*, SERPINB9, SERPINC1, SERPINE1, SERPINE2*, SERPINF2, SERPING1*, SERPINH1, TIMP1*	
**Membrane receptors**			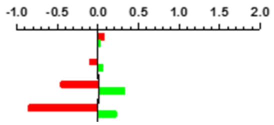
**Immune and cytokines**	5	FAS, FCGRT, IL17RA, TLR2*, TLR3*	
**Hormones and Growth factors**	5	EGFR, GPR5C, IGF2R, M6PR, MET	
**Cell-cell adhesion and communication**	4	ANTXR2, CXADR, EPHB4, PTPRF	
**(micro)nutrient**	3	ASGR1, LSR, TFRC	
**Membrane and canalicular transporters**			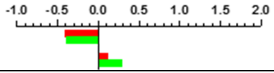
**ABC**	4	ABCB1, ABCC1#, ABCC2, ABCC3	
**SLC**	7	SLC16A1, SLC16A7*, SLC22A18*, SLC29A1, SLC22A6*#, SLCO2B1, SLCO1B1*	
**Tight junctions**			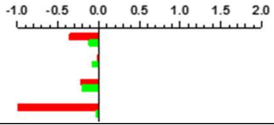
**MAGUK and protein kinase**	4	CASK, PRKCI, ZO-1, ZO-2	
**Occludin, Claudin and Immunoglobulin**	3	CLDN1, F11R (JAM), OCLN	
**Ezrin/radixin/moesin**	3	EPB41, EZR, RDX	
**Morphogenesis**	5	CGN*, CTNNB1*, MLLT4*, SPTBN1, TJAP1	
**Polarisation**			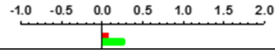
**Rho, PAR3, lin-7, LAP, and MAGUK**	6	CDC42, DLG1*, LIN7A, LIN7C, PARD3, SCRIB	
**Cytoskeleton**			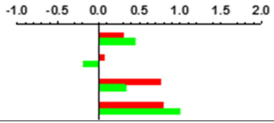
**Actin-associated**	11	ACTB, ACTG1, ACTN1, ACTN4, ACTR2*, ACTR3, ACTR10, CFL1, CFL2, VASP, VCL	
**Myosin and tropomyosin**	11	MYH9, MYH10, MYL1*, MYL6, MYL12A, MYL12B, MYLK, MYO19, MYO1E, TPM3, TPM4	
**Tubulin-associated**	15	TBCA, TBCB, TBCE, TPPP*, TUBA1A*#, TUBA1B*, TUBA1C, TUBA4A, TUBB, TUBB2A, TUBB3, TUBB4, TUBB6, TUBG, TUBGCP2	
**Other**	1	KIF2A	
**Trafficking**			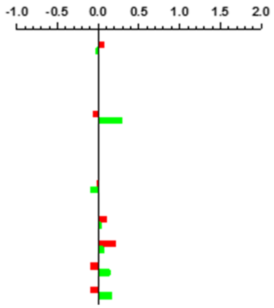
**Rho and Rho-associated**	27	ARAP1, ARHGAP1, ARHGAP12*#, ARHGAP17, ARHGAP18, ARHGAP35, ARHGAP44*#, ARHGAP5, ARHGDIA, ARHGEF1, ARHGEF10L, ARHGEF12*, ARHGEF18, ARHGEF2, ARHGEF5*, ARHGEF7, FARP1, RAC1, RAC2*, RHOA*, RHOB, RHOC, RHOG, RHOT2, ROCK1, ROCK2, SRGAP1*#	
**Rab and Rab-associated**	62	CHM, ERC1, GDI1, GDI2, RAB10, RAB11B, RAB11FIP5*#, RAB12, RAB13, RAB14, RAB18, RAB1A, RAB1B, RAB21, RAB22A, RAB23, RAB24, RAB27A, RAB27B*, RAB29, RAB2A, RAB2B, RAB30, RAB32, RAB35, RAB3A, RAB3GAP1*, RAB3GAP2, RAB43, RAB4A, RAB4B, RAB5A, RAB5B, RAB5C*, RAB6A*, RAB6B*#, RAB6C*#, RAB7A, RAB8A, RAB8B, RAB9A, RABAC1, RABEP1, RABEP2, RABEPK, RABGAP1, RABGAP1L*, RABL3, RILP, RIN1*, TBC1D1*, TBC1D15, TBC1D17, TBC1D2, TBC1D22A*, TBC1D23, TBC1D2B*, TBC1D31*#, TBC1D4, TBC1D5, TBC1D8B, TBC1D9B	
**Ras and Ras-associated**	21	DAB2IP, G3BP1, G3BP2, HRAS, IQGAP1, IQGAP2, IQGAP3, NRAS, PDE6D*, RALA, RALB, RAP1A, RAP1B, RAP2C, RASA1, RIN1*, RRAGA, RRAGC, RRAS, RRAS2, RSU1	
**Clathrins**	5	CLINT1*, CLTB, CLTC, CLTCL1, PICALM	
**SNAREs**	5	BNIP1, SNAPIN, USE1, VTI1A, VTI1B	
**Coatomer-associated**	8	COPA, COPB1, COPB2, COPE, COPG1, COPG2, COPZ1, COPZ2*	
**Others**	3	GOLT1B, SFT2D2, SFT2D3	
**Degradation machineries**			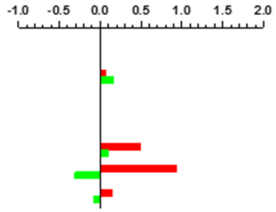
**Ubiquitin-proteasome system**	77	ADRM1, BAG2, BAG3, BAG5, BAG6, CACUL1*, CAND1, CUL1, CUL2, CUL3, CUL4A, CUL4B, CUL5, DDI2, DNAJB2, ECM29, PAAF1, PSMA1, PSMA2, PSMA3, PSMA4, PSMA5, PSMA6, PSMA7, PSMB1, PSMB10, PSMB2, PSMB3, PSMB4, PSMB5, PSMB6, PSMB7, PSMB8, PSMB9, PSMC1, PSMC2, PSMC3, PSMC4, PSMC5, PSMC6, PSMD1, PSMD6*, PSMD10, PSMD11, PSMD12, PSMD13, PSMD14, PSMD2, PSMD3, PSMD4, PSMD5, PSMD6*, PSMD7, PSMD8, PSMD9, PSME1, PSME2, PSME3, PSME4, PSMF1, PSMG1, PSMG2, PSMG3, PSMG4, RAD23A, RAD23B, RBX1, UBE3A, UBE3C, UBQLN1, UBQLN4, UBR1, UBXN1, UCHL5, USP14, USP47, VCP	
**Cathepsins**	8	CTSB, CTSD, CTSF*, CTSH, CTSL, CTSO*, CTSS*, CTSZ*	
**Calpains (CAPNs)**	3	CAPN1, CAPN2, CAPNS1	
**COP9 signalosome complex**	8	COPS2, COPS3, COPS4, COPS5, COPS6, COPS7A, COPS7B, COPS8	

The comparison of protein abundances (copy number per cell) was made using the HepaRG, primary human hepatocyte (PHH), and HepG2 proteomics datasets. Only the proteins commonly quantified in at least two of the three systems were considered for this comparison. For a given family or system, the mean fold change of copy numbers of proteins commonly quantified in the three systems is shown in red (HepaRG vs. PHH) and green (HepG2 vs. PHH). *indicates proteins that are not present in the HepG2 proteomics dataset. # indicates proteins that are not present or for which quantitative values are not available in the PHH proteomics dataset. See supplemental Information for detailed annotations of proteins.
